# Engineering the flagellar type III secretion system: improving capacity for secretion of recombinant protein

**DOI:** 10.1186/s12934-019-1058-4

**Published:** 2019-01-18

**Authors:** Charlotte A. Green, Nitin S. Kamble, Elizabeth K. Court, Owain J. Bryant, Matthew G. Hicks, Christopher Lennon, Gillian M. Fraser, Phillip C. Wright, Graham P. Stafford

**Affiliations:** 1 Integrated BioSciences, School of Clinical Dentistry, University of Sheffield, Sheffield, S10 2TA UK; 2 School of Engineering, The Faculty of Science, Agriculture and Engineering, Newcastle University, Newcastle, NE1 7RU UK; 3 Department of Pathology, University of Cambridge, Cambridge, CB2 1QP UK; 4 FUJIFILM Diosynth Biotechnologies, Belasis Avenue, Stockton-on-Tees, Billingham, TS23 1LH UK; 5 Sustainable Process Technologies, Chemical and Environmental Engineering, University of Nottingham, Nottingham, NG7 2RD UK

**Keywords:** Recombinant protein secretion, Flagellar, Strain engineering, Secretion assay, Biotechnology, Synthetic biology

## Abstract

**Background:**

Many valuable biopharmaceutical and biotechnological proteins have been produced in *Escherichia coli*, however these proteins are almost exclusively localised in the cytoplasm or periplasm. This presents challenges for purification, i.e. the removal of contaminating cellular constituents. One solution is secretion directly into the surrounding media, which we achieved via the ‘hijack’ of the flagellar type III secretion system (FT3SS). Ordinarily flagellar subunits are exported through the centre of the growing flagellum, before assembly at the tip. However, we exploit the fact that in the absence of certain flagellar components (e.g. cap proteins), monomeric flagellar proteins are secreted into the supernatant.

**Results:**

We report the creation and iterative improvement of an *E. coli* strain, by means of a modified FT3SS and a modular plasmid system, for secretion of exemplar proteins. We show that removal of the flagellin and HAP proteins (FliC and FlgKL) resulted in an optimal prototype. We next developed a high-throughput enzymatic secretion assay based on cutinase. This indicated that removal of the flagellar motor proteins, *motAB* (to reduce metabolic burden) and protein degradation machinery, *clpX* (to boost FT3SS levels intracellularly), result in high capacity secretion. We also show that a secretion construct comprising the 5′UTR and first 47 amino acidsof FliC from *E. coli* (but no 3′UTR) achieved the highest levels of secretion. Upon combination, we show a 24-fold improvement in secretion of a heterologous (cutinase) enzyme over the original strain. This improved strain could export a range of pharmaceutically relevant heterologous proteins [hGH, TrxA, ScFv (CH_2_)], achieving secreted yields of up to 0.29 mg L^−1^, in low cell density culture.

**Conclusions:**

We have engineered an *E. coli* which secretes a range of recombinant proteins, through the FT3SS, to the extracellular media. With further developments, including cell culture process strategies, we envision further improvement to the secreted titre of recombinant protein, with the potential application for protein production for biotechnological purposes.
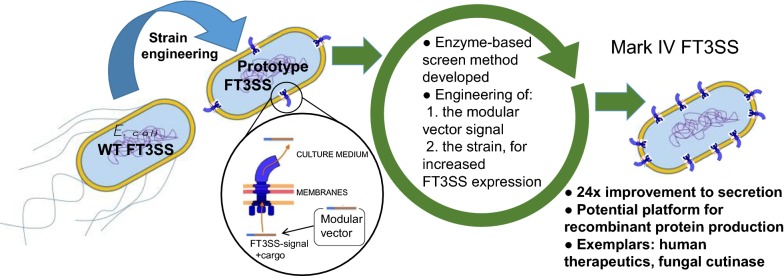

**Electronic supplementary material:**

The online version of this article (10.1186/s12934-019-1058-4) contains supplementary material, which is available to authorized users.

## Background

A persistent goal of biotechnology is to produce recombinant protein at reduced cost, while maintaining high product quality and yield. For many protein biologics, production in prokaryotes (chiefly in *E. coli*) is favoured. Major advantages include fast growth to high cell densities on cheap carbon sources, and simple scale up [1, 2]. However, as in many industrial biotechnology (IB) processes, downstream processing accounts for a high proportion of costs [3–5]. If protein product was secreted by the expression organism into the growth media, these costs could be reduced, as purification could be simplified, i.e. eliminating the need to lyse cells, or to remove cellular contaminants, which can compromise product purity, and may elicit an immune response in humans (e.g. lipopolysaccharide in *E. coli*) [6–8]. In addition, product yield and quality may be increased, as secretion would bypass cytoplasmic and periplasmic proteases. Extracellular secretion may also improve protein folding and solubility, while also reducing the formation of inclusion bodies, thus increasing product yield and quality [9–12].

Secretion of heterologous protein in *E. coli* has been utilised, however the majority of work concerns secretion into the periplasm via Sec or Tat-dependent systems [10]. While this strategy can yield titres in the mg L^−1^ range (e.g. 30 mg L^−1^ human growth hormone [13], 60 mg L^−1^ GFP [14]), this is only achieved following purification from the periplasm. Alternatively extracellular localisation of protein can be achieved In *E. coli* without directed secretion to the extracellular space via strategies that make use of ‘leaky strains’ [15], where heterologous proteins are fused to a secretion tag (for example *ompA, ompC, pelB* [16, 17]), to enable directed secretion to the periplasm, followed by leaking into the supernatant. While titres may be favourable, the presence of additional periplasmic proteins adds complexity to purification, e.g. while 550 mg L^−1^ cell culture of lipase B tagged with the *pelB* signal sequence was found in the extracellular fraction of *E. coli*, high concentrations of periplasmic proteins were also detected, and only one-fifth of the product was able to be recovered [18]. To date, true secretion (i.e. to the extracellular space via a defined secretion system, rather than through leaking or lysis) of heterologous protein by prokaryotes has been achieved at up to approximately 50 mg L^−1^, in organisms such as *Bacillus* and *Pseudomonas* [19–21], as opposed to the g L^−1^ titres achieved in eukaryotes such as *Pichia pastoris* [22]. In *E. coli,* attempts to secrete proteins directly into the media have been limited, with evidence of low levels (i.e. 1 µg to 1 mg L^−1^ of heterologous protein) via type I secretion system (T1SS) using either the Haemolysin or lipase transporting ABC transporter systems [23–28]. Another option for direct secretion into the media, is the bacterial flagellar type III secretion system, which has been utilised in *E. coli* [29–32]*, Bacillus* [20] and *Salmonella enterica* sv. Typhimurium [33–36]. In addition, secretion of up to 30 mg L^−1^ of a range of polymers [37], such as tropo-elastin and spider silk, have also been reported using the injectisome type III secretion system of *S. enterica* [37–39].

In this report we focus on the *E. coli* FT3SS, due to a preference in IB for both *E. coli*, and non-pathogenicity associated secretion systems. *E. coli* have 4–10 flagellum of around 20 μm in length, each comprising of up to 30,000 flagellin (FliC) monomers [40–43]. The FT3SS, despite having evolved to build a flagellum and provide motility to the cell, is effectively an efficient protein secretion machineable to assemble a multi-component structure composed of several thousand subunits on its surface. Proteins initially assemble at the inner membrane, resulting in a pore of about 2.0 nm diameter, through which the majority of the remaining flagellar proteins are exported, unfolded, to the distal end of the existing flagellar structure [44]. Therefore in the context of biotechnology, the FT3SS may provide a one step, high capacity route for protein export.

The basic flagellar structure is comprised of the basal body (motor and secretion apparatus), hook (universal joint), and filament (propeller), which are assembled in an ordered manner, controlled by well-understood checkpoints [45]. One key feature is the existence of a flagella master regulator (FlhD_4_C_2_-class I) that activates class II flagellar genes. These class II genes transcribe the basal body and FT3SS apparatus, along with the sigma factor FliA, which in turn promotes the transcription of the class III genes (for the filament, motor, hook associated and hook proteins, along with the chaperone and chemotaxis proteins) [46, 47]. Furthermore, transcription is also coupled to assembly, as levels of FliA are modulated by the anti-sigma factor (FlgM), which is secreted upon hook-completion [48–50]. Additionally, given the energetic cost of motility, expression of the master regulator is modulated by many environmental cues that alter gene expression of the *flhDC* operon or activity of FlhD_4_C_2_ [51, 52].

Here, we aimed to capitalise on the fact that upon mechanical shearing or breakage, flagellar continue to secrete unfolded FliC monomers which are transported to the distal growing tip, allowing regrowth under the FliD cap [53–55]. In addition, in the absence of the FliD cap, or other structural proteins (e.g. FlgKL), secretion of FliC monomers into the media without polymerisation occurs [56, 57]. In addition, the N-terminal secretion signals of FT3SS subunits are well characterised and have been exploited to direct non-FT3SS subunits for secretion through the FT3SS in *E. coli* [29–32] and (their close relatives) *Salmonella* [34–36]. We aimed to develop an improved, modular FT3SS *E. coli* strain for high yielding secretion of a range of therapeutic recombinant proteins. Our eventual aim is to surpass the secretion titres reported in other *E. coli* secretion systems, by achieving extracellular secreted titres in the high mg L^−1^ range. We hope to accomplish this using controlled secretion to the extracellular space, combined with the absence of cellular contaminants. Here we report progress towards these targets via strain development, secretion signal optimisation, rigorous use of controls, and development of a high-throughput assay to measure FT3SS secretion.

## Results

### Establishing a prototype FT3SS secretion strain and defining optimal parameters for protein expression and secretion

**Fig. 1:**
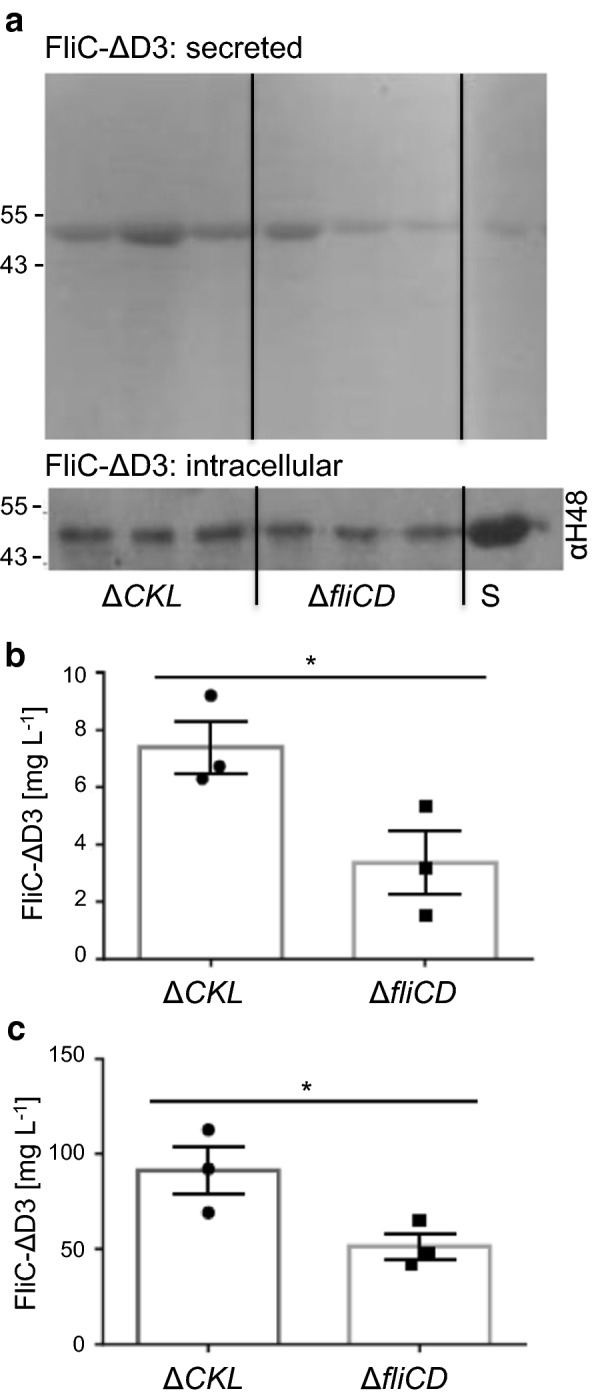
Comparison of secretion capacity in the HAP-less and cap-less strains. *E. coli* MC1000 Δ*fliC* Δ*flgKL* (ΔCKL) and Δ*fliC*D containing the plasmid pTrc-FliC-ΔD3 were grown in LB supplemented with 0.05 mM IPTG, and harvested at OD_600_ 1.5. Samples which represented either 25 µL or 400 µL culture media were loaded for SDS-PAGE, for intracellular and secreted protein respectively. **a** Representative images showing the secreted fractions following Coomassie staining, and the intracellular fractions following immunoblotting using an anti-flagellin (H48) antibody and a HRP secondary. A FliC-ΔD3 protein standard (S) was also included, to allow quantification of protein concentration by densitometry (**b**, **c**). Quantifications for biological triplicates, ± SE and individual data points shown, *p < 0.05

In this study we used *E. coli* MC1000 as the parent strain, partly for biosafety given its leucine auxotrophy, but also given the presence of an IS5 insertion sequence upstream of *flhD.* This reduces negative transcriptional regulation on *flhDC,* by disrupting promoter binding sites for the negative regulators LrhA and OmpR (for annotated sequence see Additional file 1: Fig. S1) [58–60]. Previous studies of FT3SS dependent protein secretion in *E. coli* employed a Δ*fliD* ‘cap-less’ strain [29–31, 36]. We also considered alternative strategies, given the potential for this strain to influence and suppress FlhD_4_C_2_ activity via post-translational protein–protein interactions of the FlhDC complex with FliT (the chaperone of FliD) [61, 62]. Specifically, we created a ‘HAP-less’ (Δ*flgKL)* strain, which would not incur feedback on FlhD_4_C_2_^−^, but would theoretically decouple secretion from assembly, i.e. as opposed to the Δ*fliD* strain where more free FliT would potentially repress FlhD_4_C_2_ activity, and thus flagellar gene expression [61]. In addition, it has also been established that in Δ*flgKL* mutants, unpolymerised FliC monomers are secreted into the media, rather than assembling into a filament [56]. To lessen competitive secretion, strains also lacked *fliC.* We found that the HAP-less and cap-less strains displayed identical growth curve parameters (Additional file 1: Fig. S2). We then compared their ability to export a 39 kDa shortened version of FliC that lacked the central D3 domain (residues 191–283) so that it could be easily distinguished from native FliC. This secretion substrate was termed FliC-ΔD3 and was inserted into plasmid pTrc99a-FF to create pTrc-FliC-ΔD3, and expressed by addition of IPTG. This substrate was efficiently secreted (Fig. 1) and also produced a fully motile strain when provided *in trans* in a Δ*fliC* strain (Additional file 1: Fig. S3).

As shown in Fig. 1a, b when FliC-ΔD3 was expressed with induction using 0.05 mM IPTG, we observed approximately twofold more FliC-ΔD3 (on average 7.4 mg L^−1^) in the secreted fraction of the HAP-less strain in comparison to the cap-less. Notably, we also observed increased amounts of FliC intracellularly in the HAP-less strain (Fig. 1a, c) but found overall that the HAP-less strain was 1.3 times more efficient at secreting protein (secreted FliC-ΔD3 as a function of total FliC-ΔD3, i.e. 7.4 mg L^−1^ divided by 98 mg L^−1^ for the HAP-less strain). Based on the absence of additional proteins in the cell supernatant (Fig. 1a—Coomassie), we infer that the improved titre of secreted FliC-ΔD3 was due to FT3SS secretion, as cell lysis was not prevalent in either of these strains. We therefore used the HAP-less, Δ*fliC* Δ*flgKL* strain in subsequent experiments as our prototype FT3SS secretion strain.

**Fig. 2:**
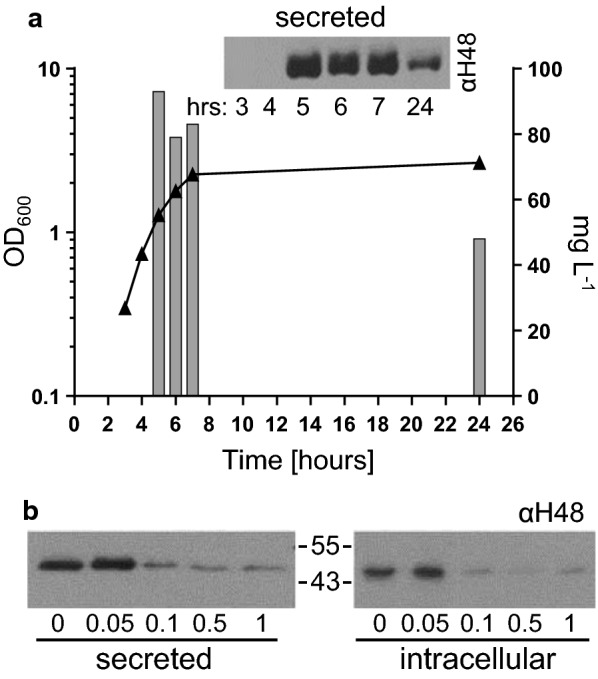
Optimisation of cell culture induction and harvesting procedure for the FliC-ΔD3 protein secretion assay. The ‘prototype’ *E. coli* MC1000 Δ*fliC* Δ*flgKL* (ΔCKL) containing the plasmid pTrc-FliC-ΔD3 was grown in 100 mL flask cultures in LB and either **a** supplemented with 0.05 mM IPTG and harvested every hour or **b** supplemented with 0, 0.05, 0.1, 0.5 or 1 mM IPTG and harvested at OD_600_ 1.0. FliC-ΔD3 was detected by immunoblot as described in Fig. 1a. Samples which represented either 25 µL or 300 µL culture media were loaded for SDS-PAGE for intracellular and secreted protein respectively. Densitometry analysis allowed quantification of secreted protein, throughout the growth curve. This was repeated to ensure that this corroborated with the general trend

Before further experimentation, we defined optimal parameters for expression and production of proteins via the FT3SS-assessing that the optimal OD_600_ for harvest of cells for maximal secretion of FliC-ΔD3, is between an OD_600_ of 1 and 2, i.e. the mid-late exponential phase (Fig. 2a and inset). We then examined the effects of varying the inducer (IPTG) concentration on expression and secretion of FliC-ΔD3. We harvested cells at an OD_600_ of 1.0 and show that 0.05 mM IPTG produced the highest concentration of both intracellular and secreted FliC-ΔD3 (Fig. 2b), therefore this procedure forms the basis of experiments.

### Preliminary improvement of the FT3SS prototype using native substrates

**Fig. 3:**
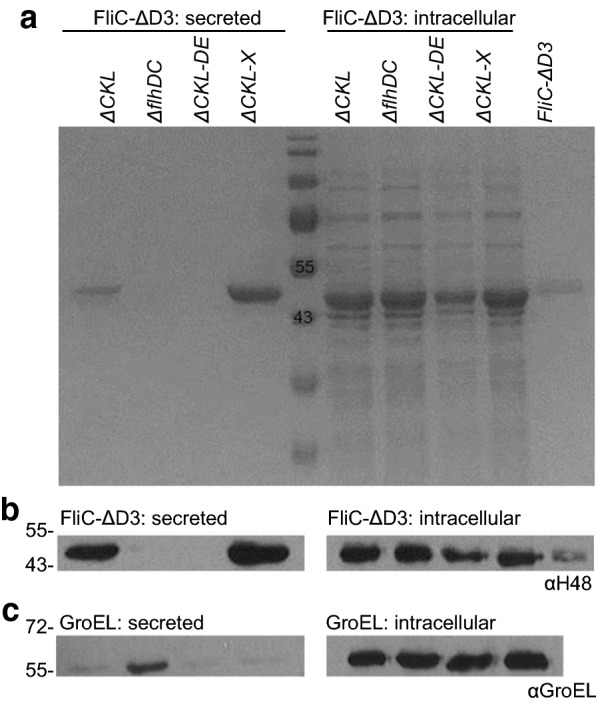
Protein secretion through the truncated FT3SS can be both controlled and improved. *E. coli* MC1000 Δ*fliC* Δ*flgKL* (ΔCKL or ‘prototype’), Δ*flhDC,* Δ*fliC* Δ*flgKL* Δ*flgDE* (ΔCKL-DE) or Δ*fliC* Δ*flgKL* Δ*clpX* (ΔCKL-X or ‘Mark II strain’) containing pTrc-FliC-ΔD3 was grown with 0.05 mM IPTG (or 1 mM to allow overexpression in Δ*flhDC*) and harvested at OD_600_ 1.0. Secreted and intracellular cell fractions were loaded for SDS-PAGE in the quantities described in Fig. 2. A FliC-ΔD3 protein standard was included to allow quantification. Samples underwent **a** Coomassie staining or immunoblot analysis of cells and supernatant using **b** anti-flagellin (αH48) or **c** anti(α)-GroEL primary antibodies and a HRP-conjugated secondary

To establish whether secretion via the FT3SS could be improved from this basal level, we considered a series of mutations that might boost overall flagellar gene expression. We then compared their effect on secretion to a number of negative controls, to establish the absolute specificity of this system. As seen in Fig. 3a, b, use of an *flhDC* null mutant, with no FT3SS present, resulted in complete lack of FliC-ΔD3 secretion even when overproduced in the cytoplasm (by addition of 1 mM IPTG). In parallel we also tested secretion of FliC-ΔD3 in a (Δ*fliC* Δ*flgKL*) ∆*flgDE* strain, in which secretion of ‘late’ filament substrates (i.e. FliC) is abrogated due to the absence of substrate specificity switching [57, 63], and observed a complete lack of secretion in this strain. Further to these controls, we also tested for the presence of the strictly cytoplasmic, GroEL chaperonin protein. Aside from a negligible amount in the *∆flhDC* strain, the cytoplasmic contaminant GroEL was absent in secreted fractions (Fig. 3c). This demonstrates that cell lysis was not prevalent, and that the two secretion strains are no more ‘leaky’ that the negative control strains. Together, this confirmed that the presence of FliC-ΔD3 monomers in the secreted fraction was due to targeted FT3SS secretion alone.

We then examined the effect of mutations that might upregulate flagellar gene expression. Our initial target was the protein quality control protein, ClpX, a component of the ClpXP protease complex which acts on FlhD_4_C_2_ [64]. In previous studies the removal of *clpX* in *Salmonella* and enterohaemorrhagic *E. coli* resulted in an accumulation of FlhD_4_C_2_, an increase in the amount of FliC subunits secreted, and increased cell motility [65–67]. Here we show that the removal of *clpX* from wildtype *E. coli* MC1000 cells resulted in an increase in filament proteins and increased motility (Additional file 1: Fig. S4a–d). The removal of *clpX* from the prototype strain resulted in an increase in both intracellular and secreted FliC-ΔD3 (Fig. 3a, b). The secretion efficiency (secreted protein as a proportion of the total (intracellular and secreted) protein) did not alter, however we did measure a 1.6-fold average increase in the titre of secreted protein, and a maximum estimated secretion of 34 mg FliC-ΔD3 L^−1^ culture medium achieved from Δ*fliC* Δ*flgKL* ∆*clpX* (calculated by comparison to a FliC-ΔD3 standard). We also noted that Δ*fliC* Δ*flgKL* Δ*clpX* cells were on average 1.2 times longer (p < 0.001. t-test) than Δ*fliC* Δ*flgKL* cells, with a higher abundance of very long cells (> 0.8 µm) (Additional file 1: Fig. S4e–g). As we detected significantly more (p < 0.05) FlhA in Δ*clpX* cells (Fig. 5c), this may suggest that elongated cells harbour a larger number of FT3SS basal bodies, thus allowing increased secretion, though we have no experimental evidence for this at this stage. Of note is that we also produced a Δ*fliC* Δ*flgKL* Δ*dksA* strain, which in contrast to what was anticipated based on published literature [68, 69], did not result in any noticeable increases in FT3SS secretion. As a result, we moved on with the Δ*fliC* Δ*flgKL* ∆*clpX* strain as a Mark II FT3SS strain to test additional strain modifications.

### Development of a prototype secretion plasmid and high-throughput assay to measure secretion

In order to move towards our goal of production and secretion of heterologous proteins via the FT3SS, we designed and tested a synthetic prototype modular vector. This contained elements aimed at optimal secretion of proteins via the FT3SS, alongside their subsequent detection and ultimately purification (FLAG and Streptavidin, TEV protease. See Additional file 1: Fig. S5). These were produced as plasmids in the IP-Free pJEXpress backbone (DNA2.0), with the first 47 amino acids of FliC (the secretion signal [70]), the *fliC* 5′UTR (which also harbours the native *fliC* promoter, thus allowing both T5 based-IPTG inducible, and flagellar mediated (class III, via FliA [71, 72]) gene expression), alongside the *fliC* 3′UTR, both of which have been implicated in influencing secretion through the FT3SS [30, 31]. Finally, restriction enzyme sites were incorporated throughout to allow modular modification of the secretion construct; notably *Eco*RI and *Pst*I sites allow for the insertion of any cargo gene for secretion.

We initially chose a heterologous protein target for secretion which was non-bacterial, commercially relevant and assayable. This enabled ease of screening for secretion via an activity assay, thus facilitating higher throughput screening for improved FT3SS strains. Our choice was the well-characterised 20.8 kDa cutinase from the unicellular fungus *Fusarium solani,* which had previously been expressed in *E. coli* intracellularly [73, 74], and for which a well-established activity assay based on its esterase activity is known [75]. Advantageously, cutinase is also relevant in IB processes, e.g. degradation of plastics [76, 77]. An *E. coli* codon optimised cutinase was synthesised (GeneArt^®^) and inserted into the pJex-*fliC*47-empty plasmid resulting in pJex-*fliC*47-cutinase, which is predicted to yield a 30.6 kDa peptide.

**Fig. 4:**
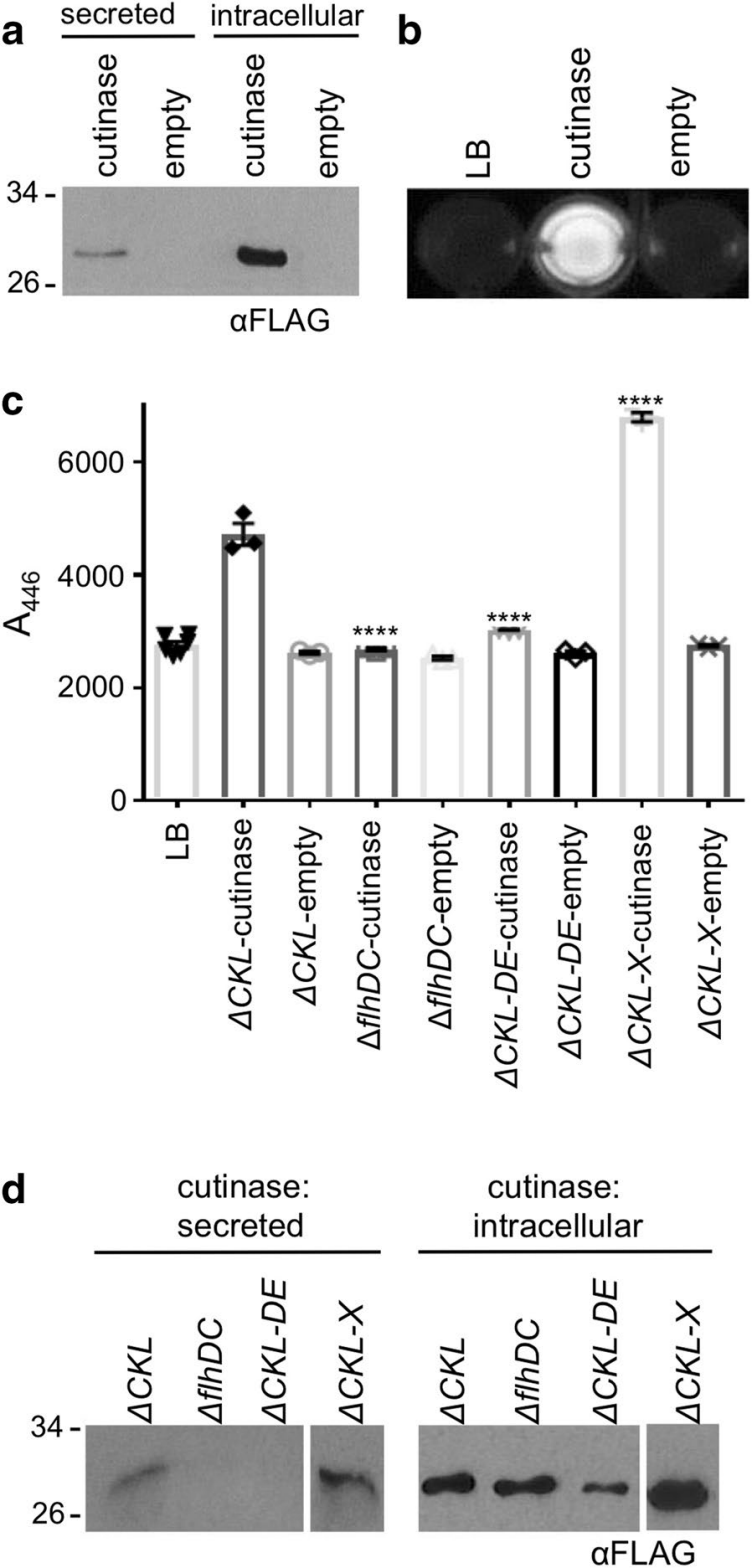
Development of a high throughput fluorescence assay to measure protein secretion through the truncated FT3SS. The ‘prototype’ *E. coli* MC1000 Δ*fliC* Δ*flgKL* (ΔCKL) containing either pJex-*fliC*47-cutinase or pJex-*fliC*47-empty were grown as described in Fig. 3 and prepared for **a** immunoblot with the anti-FLAG-HRP (αFLAG) antibody (where samples representing either 15 µL or 300 µL culture media for intracellular and secreted protein respectively were loaded onto the SDS-PAGE) (**b**) or florescence assay: 40 µL supernatant was added to 160 µL MUB substrate in a 96 well plate. Following incubation for 30 min at 30 °C, samples were visualised under UV light. **c**, **d** *E. coli* strains Δ*fliC* Δ*flgKL* (ΔCKL or ‘prototype’), Δ*fliC* Δ*flgKL* ∆*clpX* (ΔCKL-X or ‘Mark II strain’), ∆*flhDC* and Δ*fliC* Δ*flgKL* ∆*flgDE* (ΔCKL-DE) harbouring the cutinase expressing or empty vector plasmid were grown and **c** prepared for MUB secretion assay as described above; however following incubation, fluorescence was measured in a plate reader (excitation 302 nm, emission 446 nm). Results from one biological replicate, with three technical repeats. ± SE and individual data points shown. Two-way ANOVA (variables: strain and plasmid) and Tukey’s multiple comparison test: ****p < 0.001. **d** Representative immunoblot of secreted and intracellular fractions prepared from the same cell cultures

Following expression of pJex-*fliC*47-cutinase in the ∆*fliC* ∆*flgKL* strain, both intracellular and secreted cutinase were detected, via the secretion construct incorporated FLAG tag (Fig. 4a). We next developed a simple fluorimetric secretion assay, based on cleavage of 4-methylumbelliferyl butyrate (MUB) by cutinase to yield a fluorescent 4-methylumbelliferone (4-MU) [78]. Cell-free supernatant was mixed with MUB in the presence of an appropriate buffer, and release of 4-MU was measured. This could be qualitatively observed on a UV-transilluminator to allow quick visual screening (Fig. 4b). We also established conditions for a quantitative plate-reader based version of the assay (excitation 302 nm, emission 446 nm). To test the specificity of the assay, pJex-*fliC*47-cutinase was transformed into negative control strains (∆*flhDC* and Δ*fliC* Δ*flgKL* ∆*flgDE*) as in Fig. 3. After subtraction of background MUB cleavage no activity was detected in strains containing pJex-*fliC*47-empty or LB only controls (Fig. 4c). Absence of secretion was verified by immunoblotting of the same samples (Fig. 4d). Reassuringly, we observed increased concentrations of secreted cutinase (twofold higher AU) in the Mark II Δ*fliC* Δ*flgKL* Δ*clpX* strain compared to the Δ*fliC* Δ*flgKL* prototype strain. We used this assay to detect a range of concentrations of secreted cutinase (0.04–0.20 mg L^−1^), and note that the assay could detect cutinase concentrations is excess of this. With the assay established, it was possible to screen a larger number of strains and secretion signal variants, with the aim of generating a higher capacity, Mark III secretion strain.

### Use of the high-throughput cutinase screen to establish an optimised secretion strain

**Fig. 5:**
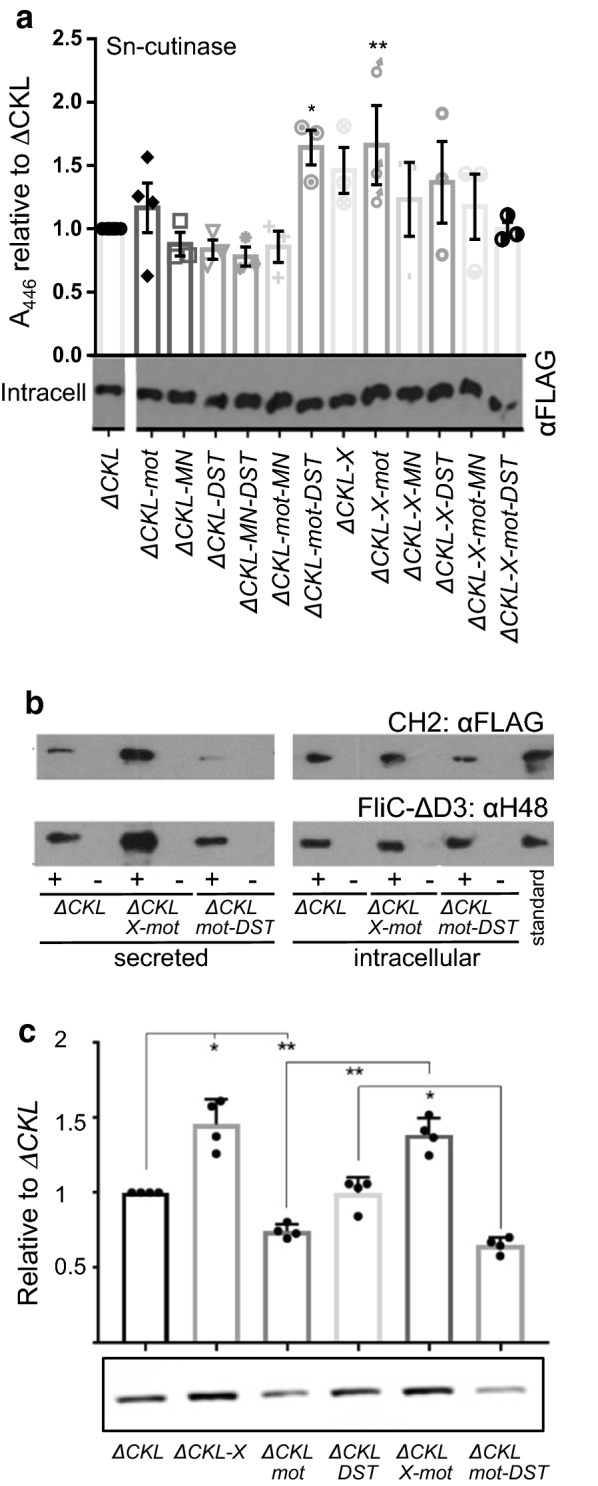
Screening for strains which are high capacity secretors of recombinant cutinase, and additional substrates. **a** The ‘prototype’ *E. coli* MC1000 Δ*fliC* Δ*flgKL* (ΔCKL) strain with additional combinations of: Δ*motAB* (mot) Δ*flgMN* (MN) Δ*fliDST* (DST) or Δ*clpX* (X), which contained pJex-*fliC*47-cutinase were grown with 0.05 mM IPTG and harvested at OD_600_ 1.0. Secreted fractions (Sn) were analysed by the MUB florescence assay as described in Fig. 4c. Results from three biological replicates were normalised to ΔCKL, ± SE shown, one-way ANOVA: p ≤ 0.001. Tukey’s multiple comparison test to ΔCKL: *p ≤ 0.05, **p ≤ 0.01. Representative immunoblot of the intracellular fraction from 15 µL cell culture shown below. **b** *E. coli* ΔCKL (‘prototype’), ΔCKL-X-mot (Mark III strain) and ΔCKL-mot-DST expressing pJex-*fliC*47-CH_2_ (+, upper), pTrc-FliC-ΔD3 (+, lower), or empty vector (−) were grown (as above) and prepared for immunoblot using either an anti-FLAG-HRP (αFLAG) antibody (upper) or an anti-flagellin (αH48) antibody and a HRP conjugated secondary (lower). The equivalent of 15 µL cell culture was loaded for intracellular fractions, and either 300 or 60 µL for the secreted fractions (for CH_2_ and FliC-ΔD3 respectively), along with the relevant protein standard to allow quantification. **c** Whole cell fractions underwent immunoblot with an anti-FlhA antibody (αFlhA), results from densitometry are presented relative to ΔCKL. Five biological repeats, ± SE and individual data points shown. One-way ANOVA: p < 0.005 and Tukey’s multiple comparison test: *p < 0.05, **p < 0.01

The MUB-cutinase assay was next utilised to assess the secretion capacity of a number of alternative FT3SS secretion strains. These were engineered to upregulate flagellar expression, or reduce metabolic burden via the deletion of redundant genes. These included the *clpX* knockout strain outlined previously, and three additional targets: ∆*flgMN* (FlgM a negative regulator of class III flagellar gene regulation, and FlgN chaperone for FlgK and regulator of FlgM [50, 79, 80]), ∆*fliDST* (FliT—an anti-FlhD_4_C_2_ factor and FliST—chaperones of FliCD [61, 81]) and ∆*motAB* (MotAB responsible for energising flagellar rotation, but not required for secretion [82, 83]). Following their construction and establishing that growth of all strains was comparable to ∆*fliC* ∆*flgKL* (Additional file 1: Fig. S6), the supernatant was analysed for the presence of cutinase via the MUB fluorescence assay (Fig. 5a). Of all the combinations tested, only ∆*fliC* ∆*flgKL* ∆*motAB* ∆*fliDST* and ∆*fliC* ∆*flgKL* ∆*clpX* ∆*motAB* resulted in a significant improvement to secretion, in comparison to the original ∆*fliC* ∆*flgKL* strain: 1.64- and 1.67-fold respectively, (p < 0.05 and < 0.01). To ensure that poor secretion was not a factor of poor cutinase expression, the presence of intracellular cutinase in all strains was confirmed with immunoblotting (Fig. 5a—intracellular).

Having identified high capacity secretion strains that could secrete active fungal cutinase, we now wished to test a human recombinant protein: an *E. coli* codon optimised version of the gene encoding human ‘CH_2_’ (CH_2_) ScFv antibody fragment (23 kDa, donated by J. Pandhal, University of Sheffield [84, 85]), which is used as an adjuvant in human antibody drugs. This was inserted into our modular secretion vector. We compared secretion of CH_2_ and the native substrate, FliC-ΔD3, in the most promising secretion strains, and observed (similarly to cutinase) improved secretion in Δ*fliC* Δ*flgKL* ∆*clpX* ∆*motAB* strain: 2.57× (CH_2_) and 3.75× (FliC-ΔD3) over that observed for the prototype strain (Fig. 5b). For both substrates this amounted to a 2.8-fold improvement to secretion efficiency. In contrast, the Δ*fliC* Δ*flgKL* ∆*motAB* ∆*fliDST* strain did not perform in a similar manner to cutinase, when secreting CH_2_ and FliC-ΔD3, with no improvement to secretion over the prototype. We thus concentrated on Δ*fliC* Δ*flgKL* ∆*clpX* ∆*motAB,* as a Mark III strain.

To gain more information on the varying success of these two strains, we used an antibody directed against the FlhA protein (which is present as part of the FT3SS export apparatus) as a proxy indicator of the number of flagellar export apparatus in the cell population. Since its gene expression is under the control of FlhD_4_C_2_, FlhA is also an indicator of total flagellar gene expression. Whole cell lysates were examined by immunoblot and quantified by densitometry analysis (Fig. 5c). Mutant strains representing the individual knockout strategies were also included as controls. Overall, these data indicate that deletion of *clpX* results in an increase in FlhA levels by approximately 1.5-fold in both the Δ*fliC* Δ*flgKL* and Δ*fliC* Δ*flgKL* Δ*motAB* backgrounds (p < 0.05). In contrast, deletion of *motAB* alone seems to reduce FlhA levels (0.75-fold; p < 0.01). Overall, it is evident that the absence of *clpX* in strains upregulates FlhA levels, and by extension, other class II and III genes, which may explain the improvements to secretion we observed.

### Improvements to secretion efficiency by secretion signal modification

**Fig. 6:**
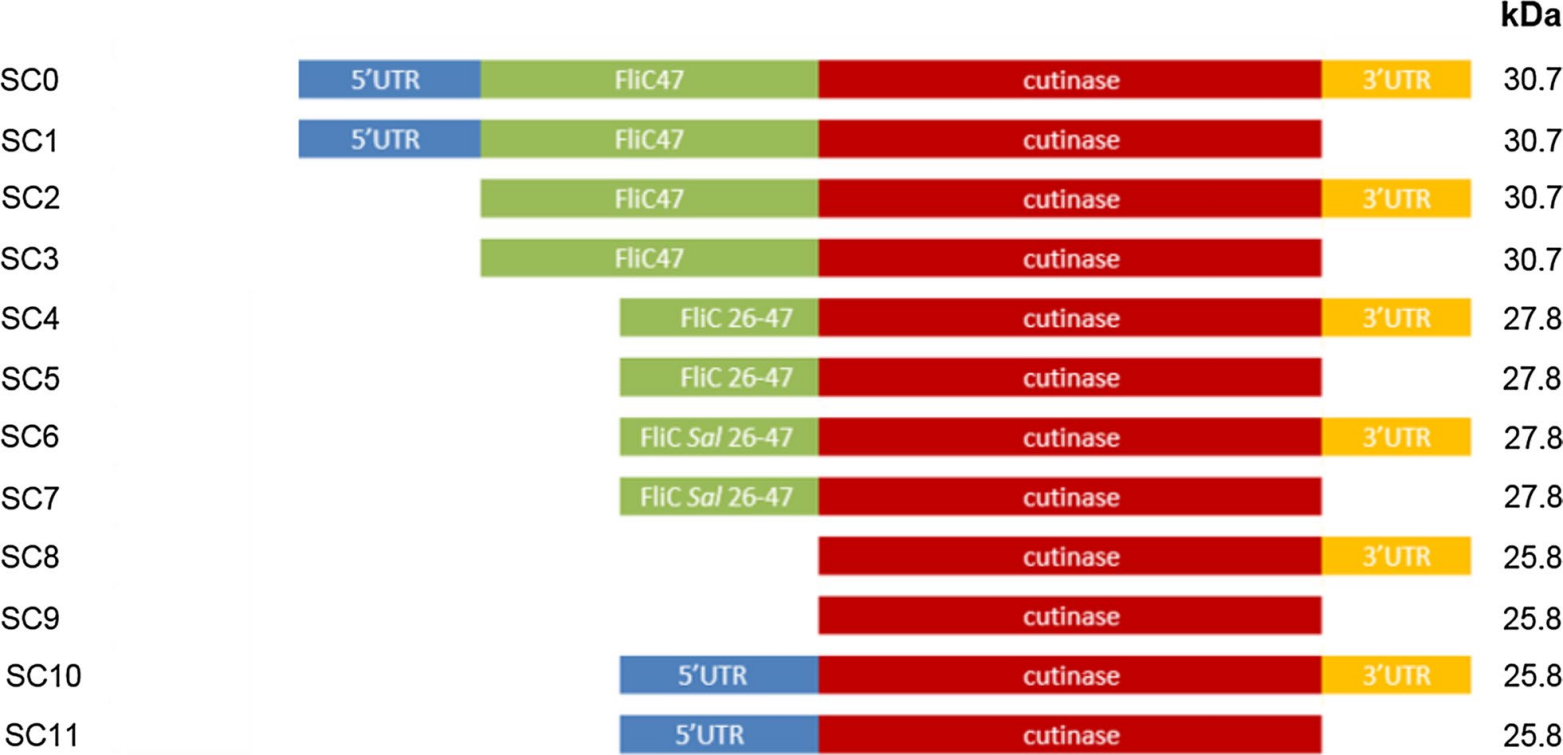
Schematic of secretion signal variants. The prototype secretion construct (SC0), and variations (SC1–SC11. Note that SC1 contributes to the ‘Mark IV’ improvement) are depicted along with the predicted size of the protein product (kDa). All secretion constructs harbour cutinase, along with combinations of the 3′UTR, 5′UTR, the 1–47 residue FliC secretion signal, or the truncated secretion signal (residues 26–47) with the native *E. coli* or *S. typhimurium* codon usage. Construction of these plasmids is outlined in Additional file 1: Fig. S5 and Table S2

**Fig. 7:**
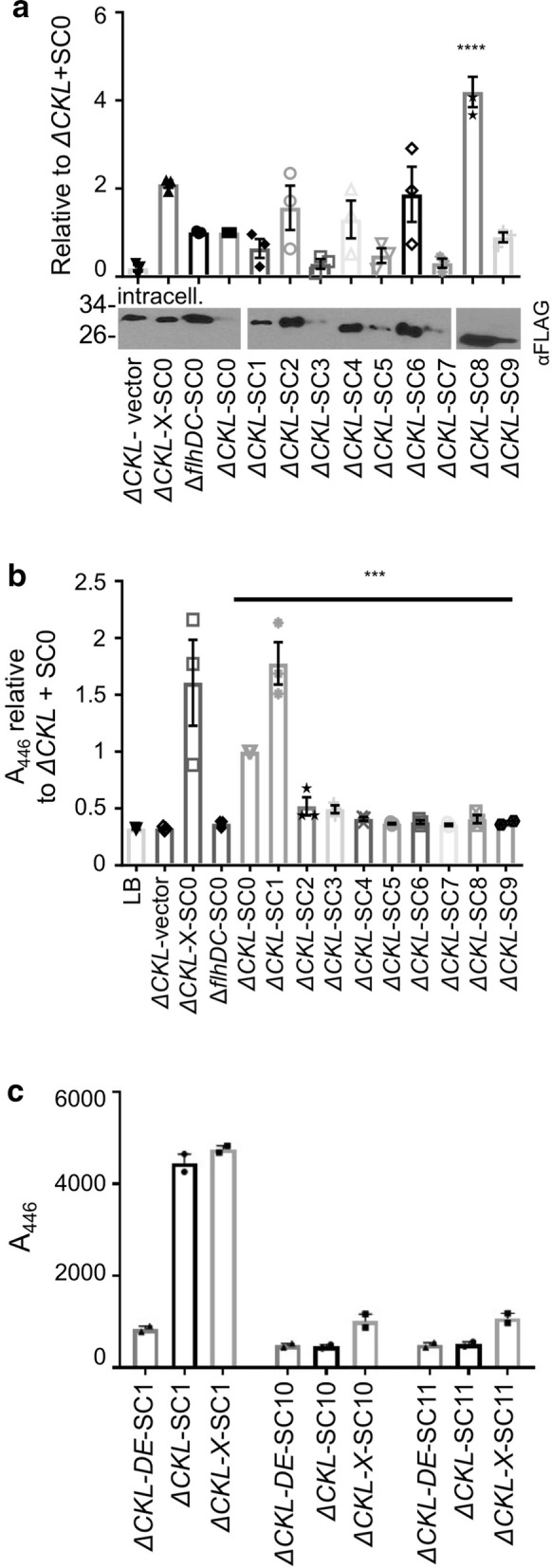
Comparison of expression and secretion in secretion signal variants. **a**, **b** The ‘prototype’ *E. coli* Δ*fliC* Δ*flgKL* (ΔCKL) harbouring either pJex-*fliC*47-empty or the secretion signal variants SC0–SC9 (see Fig. 6), along with Δ*flhDC* and *ΔfliC ΔflgKL ΔclpX* (ΔCKL-X or ‘Mark II strain’) expressing SC0 were grown and harvested as in Fig. 3. **a** Intracellular fractions were prepared for immunoblotting (plus densitometry analysis: representative image shown), and **b** secreted fractions for MUB secretion assay, as described in Fig. 4a and c respectively. Three biological repeats, normalised to ΔCKL-SC0, ± SE and individual data points shown. One-way ANOVA (for SC0–SC9 expressing strains only) p < 0.001 and Tukey’s multiple comparison test (to ΔCKL-SC0): ****p < 0.001, ***p < 0.005. **c** The absence of the 47 residue FliC signal, with the presence of the *fliC* 5′UTR was investigated in ΔCKL, ΔCKL-X and Δ*flhDC* compared to SC1. Procedure as described for Fig. 7b. Two biological replicates, ± SE and individual data points shown

In parallel to the studies aimed at strain engineering for increased FT3SS capacity, we also examined the influence of the secretion signal employed in the plasmid-based modular secretion vector. The reasoning here was that the literature reports that the inclusion of the first 47 residues of FliC [70], the 5′ and 3′UTRs [29, 30, 35], or a truncated FliC 26–47 construct with either the *Salmonella* or *E. coli* version of residues 26–28 [34, 35], can direct export of protein through a truncated FT3SS. All secretion construct (denoted SC) variants were inserted in frame with the cutinase gene, with the predicted size in kDa shown (Fig. 6). These studies were conducted using the prototype ∆*fliC* ∆*flgKL* strain, with a ∆*flhDC* strain and empty vector as negative controls. Expression of the prototype secretion construct (labelled as SC0 here) in the Mark II ∆*fliC* ∆*flgKL* ∆*clpX* strain served as an additional positive comparator. The data show that while all constructs produced cutinase (Fig. 7a), some constructs neither expressed nor secreted well (SC3, 5, 7), while others had low intracellular but significantly higher secreted levels of cutinase (SC1, p < 0.005), or, significantly high intracellular cutinase (p < 0.001) with none secreted (SC8) (see Fig. 7b). From these data it is possible to infer that the 3′UTR—while putatively influencing intracellular stability of cutinase-seems to actually reduce export (SC3, 5, 7). Whereas the intact 5′UTR and FliC 1–47 alone (SC1) seem to permit the highest levels of secretion, as this exports cutinase at 1.8-fold higher concentrations than the prototype (SC0). This led us to examine the possibility that the FliC 5′UTR alone, has the ability to direct secretion-as has been indicated by other workers [30]. However, when we inserted the cutinase gene into plasmids containing only the 5 and 3′UTRs (SC10) or the 5′UTR alone (SC11), we observed that despite good expression in all strains (Additional file 1: Fig. S7), cutinase secretion was at least fourfold lower (Fig. 7c). With this evidence, the plasmid harbouring SC1 was taken forward (for the construction of a Mark IV secretion strain) as the best candidate for increased secretion through the FT3SS.

### Confirmation that the ‘late’ FliC signal outperforms an ‘early’ hook-cap signal

As a final step to investigate the optimal secretion signal, we compared secretion of our ‘late’ FliC signal with early flagellar substrate signal sequences, namely the hook (FlgE) and hook-cap (FlgD) proteins. In order to compare these signals, we constructed cutinase harbouring secretion signal sequence variants using the first 100 amino acids of FlgD or FlgE (Additional file 1: Fig. S8a) for testing in both an early-locked, hook-less [Δ*fliC* Δ*flgKL* ∆*flgDE* (∆*clpX*)], and a substrate switched [Δ*fliC* Δ*flgKL* (Δ*clpX*)] background. The FlgE signal did not permit secretion in either strain, and the FlgD signal was four- to fivefold less effective at export than the FliC signal (Additional file 1: Fig. S8b). This indicated that despite the streamlined nature of the hook-less strain, it is in fact less efficient for secretion than the HAP-less strain. We did observe low-levels of cutinase secretion with SC13 (FlgD signal) in the Δ*fliC* Δ*flgKL* Δ*clpX* strain, presumably due to higher expression of secretion apparatus, but still at a much lower level (fourfold) than with the intact FliC signal (SC1).

### Examination of combined strain and secretion signal improvements

**Fig. 8:**
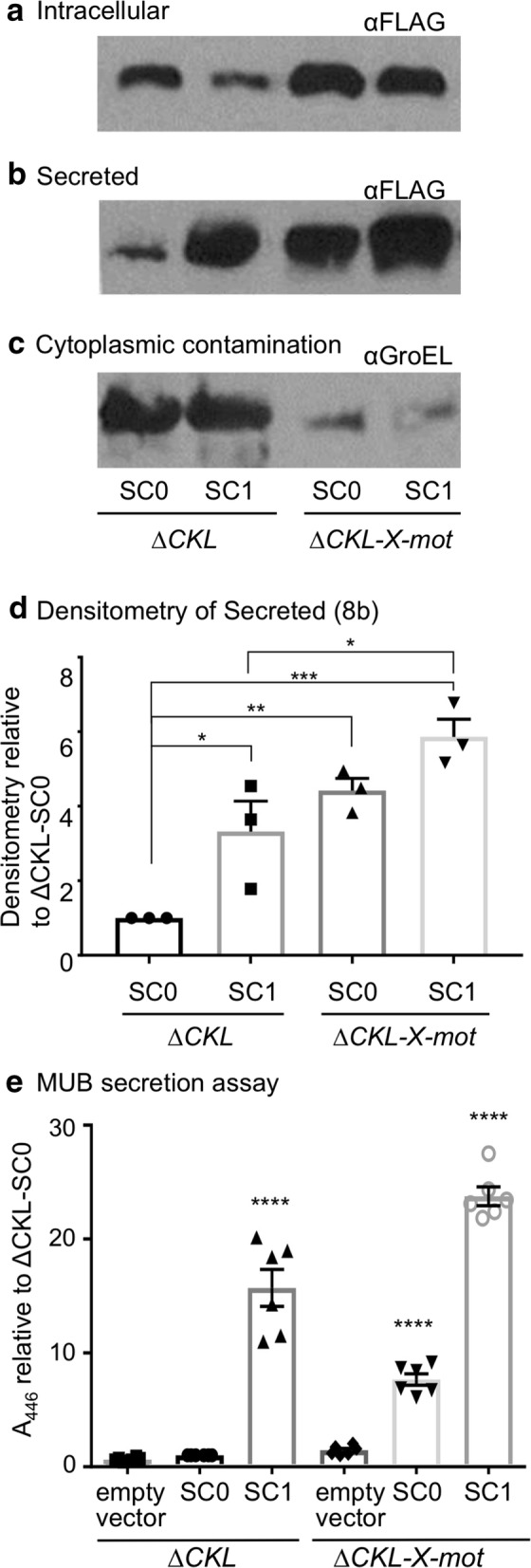
Cutinase expression and secretion: comparing the prototype, and most improved, strains and secretion constructs. The *E. coli* ‘prototype’ Δ*fliC* Δ*flgKL* (ΔCKL) or ‘Mark III strain’ Δ*fliC* Δ*flgKL* Δ*clpX* Δ*motAB* (ΔCKL-X-mot) expressing either the prototype (SC0), improved (SC1) modular secretion vector, or pJex–*fliC*47-empty (empty), were grown as described in Fig. 3. Note that ΔCKL-X-mot expressing SC1 is our ‘Mark IV strain’. Both intracellular and secreted fractions underwent immunoblot analysis using either, an anti-FLAG-HRP (αFLAG) antibody to detect **a** intracellular and **b** secreted cutinase, or **c** anti-GroEL and a HRP secondary antibody to detect cytoplasmic protein contamination. Samples representing 15 μL and 300 μL cell culture were loaded for intracellular and supernatant samples, respectively. **d** Densitometry analysis was carried out on αFLAG probed, secreted fractions (Fig. 8b: representative image) of three biological replicates, normalised to ΔCKL-SC0, ± SE and individual data points shown. One-way ANOVA, p < 0.005 and Tukey’s multiple comparison test (to ΔCKL-SC0): ***p < 0.005, **p < 0.01, *p < 0.05. **e** Supernatant was also prepared for MUB protein secretion assay as described in Fig. 4c. Six biological replicates, normalised to ΔCKL-SC0, ± SE and individual data points shown. Two-way ANOVA, p < 0.001 and Tukey’s multiple comparison test (to ΔCKL-SC0): ****p < 0.001

**Fig. 9:**
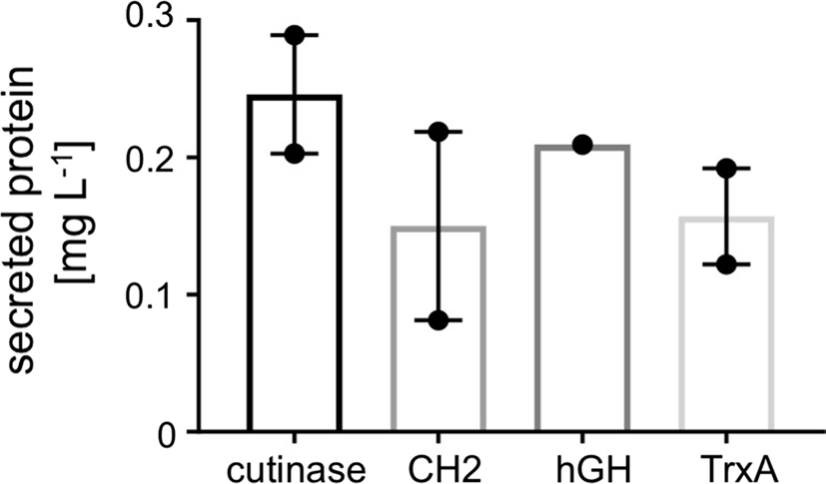
Secreted titres of a range of substrates through the optimised Mark IV secretion strain. *E. coli* Δ*fliC* Δ*flgKL* Δ*clpX* Δ*motAB* expressing plasmid based SC1 (Mark IV strains), with either cutinase, CH_2_, hGH or TrxA cargo, were grown as outlined in Fig. 3 and prepared, along with a relevant protein standard (exception: cutinase, where a hGH standard was utilised), for immunoblot detection with anti-FLAG-HRP (αFLAG). The concentration of secreted protein was then quantified by densitometry analysis. Two biological replicates (with the exception of hGH), ± SE and individual data points shown

Finally combination of the improvements to the chassis and modular secretion vector were investigated in comparison to the prototype versions. Both strains contained more intracellular cutinase following expression of SC1 over the SC0 prototype (Fig. 8a). Additionally there was more intracellular cutinase in the Mark III strain than the prototype-irrespective of secretion construct. Detection of the FLAG-tag by immunoblotting allowed visualisation of secreted cutinase by a means other than the MUB assay. In agreement with previous results (Fig. 5a, b), the Mark III strain secreted a higher concentration of substrate in comparison to the Δ*fliC* Δ*flgKL* strain when expressing the prototype secretion construct (Fig. 8b, d), this translated to a 3.83-fold improvement in secretion efficiency (Fig. 8a, b). However, the highest concentrations of secreted cutinase were seen when either strain expressed SC1, with Δ*fliC* Δ*flgKL* Δ*clpX* Δ*motAB* resulting in the highest. Given the low abundance of GroEL in Δ*fliC* Δ*flgKL* Δ*clpX* Δ*motAB* cell supernatant (Fig. 8c), we show that the increase in extracellular cutinase in this modified strain is not due to cell lysis or membrane leakage. In fact the lower level of cytoplasmic leakage in Δ*fliC* Δ*flgKL* Δ*clpX* Δ*motAB* suggests that the *clpX* allele may in fact destabilise the cell envelope, although this would require further investigation. We also note that the α-GroEL antibody is very sensitive and the Coomassie stained gel (not shown) did not indicate cell leakage in the Δ*fliC* Δ*flgKL* strain either. Using the same samples, a MUB protein secretion assay was carried out (Fig. 8e). The improvement attributed to the optimised strain (Δ*fliC* Δ*flgKL* Δ*clpX* Δ*motAB*) was 7.68-fold, and for the secretion construct (SC1) 15.70. Upon combination (Mark IV strain) these improvements were additive, resulting in a 23.78-fold increase in fluorescence in comparison to the prototypes (all p < 0.001). Densitometry analysis of secreted cutinase measured by immunoblotting (Fig. 8a, b), showed that the strain was 5.64 times more efficient at secreting cutinase than the prototype. Despite immunoblotting determining the combined fold change in the Mark IV strain to be less pronounced (Fig. 8d), the overall trend is consistent with the MUB fluorescence assay (Fig. 8e). The Mark IV strain had an average titre of 0.16 mg L^−1^ secreted cutinase (highest 0.19 mg L^−1^) (Fig. 9).

### Testing secretion of a range of recombinant proteins of human origin

Finally we investigated secretion of a number of pharmaceutically relevant, human proteins in the Mark IV FT3SS strain. These included *E. coli* codon optimised versions of, the aforementioned CH_2_ ScFv fragment, and also human growth hormone (hGH) and thioredoxin (TrxA)—both of which were kindly gifted following an ongoing collaboration with FUJIFILM Diosynth Biotechnologies, UK. Genes were PCR amplified with *Eco*RI and *Pst*I restriction site ends and inserted into the SC1 harbouring plasmid. All were secreted into the media, with respective average titres of 0.25, 0.15 and 0.21 mg L^−1^ from an OD_600_ 1.0 culture (Fig. 9, Additional file 1: Fig. S9).

## Discussion

Despite the clear benefits to protein production, to date, true secretion of recombinant protein by bacterial expression systems into the media is only possible in the mg L^−1^ cell culture range, even in high density culture [19–21]. For commercial use this requires improvement, especially when compared to eukaryotic expression organisms—such as *Pichia* which can reach 15 g L^−1^ [22]. We focus on an *E. coli* chassis, due to its preferential use in IB, for a host of beneficial reasons. In this study, we set out to engineer an *E. coli* with the ability to secrete recombinant protein into the extracellular media, in one direct step. A clear candidate for this was the one step FT3SS, and while recombinant protein has been secreted through the FT3SS previously [29–31, 34, 36], we aimed to make improvements to improve secretion capacity, without leakage, though strain engineering and design of a modular secretion and purification system.

We established that a prototype ∆*fliC* ∆*flgKL* strain outperformed a ∆*fliCD* strain (as utilised in other studies [29–31], in both protein production and secretion (Fig. 1). One explanation might be that FliD binds FliT, and so in the FliCD strain, more FliT is free to interact with, and suppress FlhD_4_C_2_ activity, thus reducing FT3SS expression and secretion [61]. In contrast removal of FlgKL frees up the chaperone FlgN to promote translation-coupled secretion of FlgM-further raising expression of FT3SS components (including the *fliC*-UTR harbouring modular secretion construct) [80], but whether this occurs in *E. coli* is not clear at present. We next investigated whether strain engineering could improve secretion, and found that removal of *clpX* (a component of the ClpXP protease complex), which is known to actively bind to, and degrade FlhD_4_C_2_ [64], improved the yield of secreted FliC by 1.6-fold, to 34 mg FliC-ΔD3 L^−1^ culture medium at OD_600_ 1.0 (Fig. 3). This became our Mark II strain. We also show total absence of FliC-ΔD3 in the secreted fractions of FT3SS null strains, along with very low abundance of cytoplasmic contamination (absence of GroEL) (Fig. 3). This observation is consistent for recombinant proteins (Fig. 4d). Furthermore we show that, in the absence of the FliC secretion signal peptide, protein is not exported (Fig. 7b, c and Additional file 1: Fig. S7). Combined, this demonstrates that in our system, the truncated FT3SS shows excellent selectivity and that leakage is not prevalent.

Development of a high-throughput, enzyme based, secretion assay (Fig. 4) enabled us to screen a range of engineered strains and secretion signal constructs (Figs. 5, 7). As enzyme activity requires correct protein folding, the assay also highlights that secreted heterologous cutinase correctly folds in the extracellular media, thus allowing functionality. This is very attractive when considering the proposed biotechnological applications of this truncated FT3SS secretion platform. Through this screen we established the ∆*flgKL* ∆*fliC* ∆*clpX* ∆*motAB* as the superior (Mark III) secretion strain (Fig. 5). Several lines of evidence supported that *clpX* deletion has a positive effect on secretion through the FT3SS [64–67]. Further to this, we observed increased filament production and motility in Δ*clpX* cells in a wild-type background (Additional file 1: Fig. S4), suggesting increased flagellar gene expression. We confirmed this notion in our truncated secretor strain, as more FlhA was detected in Δ*clpX* mutants (Fig. 5c), suggesting an increase in the number of basal bodies (secretion apparatus). In contrast, the concentration of FlhA was not increased in *motAB* mutants, indicating that improved secretion in these strains was not due to higher abundance of secretion apparatus. A probable explanation is that this strain has reduced drain on the proton motive force [82, 83], which would usually provide energy to drive flagellar rotation [86, 87], but also has implications for secretion and growth.

We also established, surprisingly, that combining the *fliDST* mutation with the Mark III ∆*flgKL* ∆*fliC* ∆*clpX* ∆*motAB* strain increased secretion of cutinase, but not other proteins (Fig. 5b), indicating that there may be some protein specific factors in determining secretion capacity. In fact the removal of *fliDST,* or *flgMN* were generally not beneficial to secretion. This may be attributed to the fact that there are contrasting reports on the effect of FlgM(N) deletion on flagellar expression in the literature [50, 79]. This is complicated by the fact that, FliS also binds FlgM prior to *fliA* expression, suppressing secretion of FlgM upon hook-basal body completion [88], while removal of FliDST per se has been shown to result in an increase in secreted FlgM [89]. In addition, FlgN is also implicated here, as a positive regulator of FlgM translation [80], further complicating this picture, and making these data both hard to predict and interpret.

We show that a construct containing the 5′UTR and FliC1–47 secretion signal (SC1) was the most efficient for secretion of recombinant proteins (Fig. 7b). In agreement with previous work [30, 33], we did not find inclusion of the 3′UTR to be necessary for secretion—as an extension to this, we also show that exclusion of the 3′UTR dramatically increases secretion, despite potentially reducing internal levels (Fig. 7a). An explanation may be linked to the evidence that the 3′UTR influences levels and stability of the *fliC* mRNA [90], which in turn has been shown to reduce FlgM secretion [67]. If this is affected by the 3′UTR region specifically, then this could explain improved secretion in the absence of the 3′UTR, as FlgM might be more readily secreted from the FT3SS—however at this time we have no evidence to support this theory. In contrast, the 5′UTR and FliC1–47 are able to both direct secretion and ensure adequate protein expression. One reason for this might be that the signal peptide confers some level of solubility or reduces formation of inclusion bodies—though again we have not tested this directly. Notably, our evidence (Fig. 7b, Additional file 1: Fig. S7) does not match that of other reports where the FliC 5′UTR [30, 31], or 26–47 residues of the FliC signal alone [34, 35], direct efficient secretion in *E. coli.* Neither does it match evidence that the 5′UTR is not necessary for secretion [35], and we can only attribute these differences to potential variances in strain background or experimental conditions. It would seem that modification of the secretion signal had a larger effect on secretion capacity than the strain improvements (15.70- and 7.68-fold improvements respectively). Most compelling, was our finding that upon combination, these improvements have an additive effect on the secretion capacity of the FT3SS (Fig. 8). The overall 23.78-fold improvement to cutinase secretion through the FT3SS is a step-forward in terms of moving towards an *E. coli* strain capable of high capacity secretion of recombinant protein into the extracellular media, in one step.

In our lab, the best secreted yield of protein achieved (with cell density OD_600_ < 1.5) in our truncated FT3SS (maximum of 240 mg L^−1^, average 80–90 mg L^−1,^ of FliC-ΔDE3 secreted into the extracellular media), surpasses that reported for alternative *E. coli* systems, which also enable secretion into the media. For example in the T1SS: typically in the µg L^−1^ range, but up to 3 mg^−1^ OD unit cell culture at high cell density [23–28, 91] and FT3SS (Δ*fliCD*): up to 12 mg L^−1^ (although under unspecified high density cell culture conditions, [30]). We also exceed reported yields achieved for secretion into the *E. coli* periplasm by the type II secretion system at low cell density (30–60 mg L^−1^ [13, 14]), without the requirement for periplasmic extraction or the risk of proteolytic enzymes compromising protein quality.

However as we are proposing the use of our strain as a secretion platform in a biotechnological context, we finally set out to investigate the performance of the Mark IV strain when secreting a broad range of industrial and pharmaceutically relevant recombinant proteins. At low cell density (OD_600_ 1.0) we achieved average yields of 0.16, 0.25, 0.15 and 0.21 mg L^−1^ for cutinase, a CH_2_ ScFv fragment, human growth hormone and thioredoxin respectively (Fig. 9). The secreted titres we report for heterologous protein are lower than those which we measure for native protein (FliC-ΔDE3). This was anticipated, as aside from a small truncation, FliC-ΔDE3 is a natural substrate of the FT3SS, whereas the heterologous proteins are non-native to both the organism and the secretion system. We acknowledge that our titres of heterologous protein are lower compared to other bacterial systems (where up to 50 mg L^−1^ was achieved) [20, 21]. However our results are still compelling, given that (unlike the majority of literature examples) we have achieved these titres from (1) culture supernatant, (2) at low cell density in *E. coli*, and (3) showed excellent control of secretion to the extracellular space, with no evidence for cell leakage. When comparing our final Mark IV strain to other strains that meet the majority of these credentials, our current strain secretes around four times less heterologous protein than the best titre reported in the T1SS (cell density unclear) [27], and around 50 times less than an alternative FT3SS system (carried out at a higher cell density and with different proteins) [30].

To match these reported titres in the future, we aim to further boost the titres of secreted protein, with a modified cell culture strategy and more strain engineering. For example, while our supernatant was harvested at a maximum cell density of OD_600_ 1.5; it is noted that transfer to high cell density culture (OD_600_ > 180) can greatly improve yields [14]. Indeed we have begun to test these strains at higher cell density in bioreactors with no obvious growth defects (unpublished data). Finally, the modular secretion vector has not only been optimised to enable high levels of expression (with the removal of the 3′UTR), and flexibility of protein cargo, but also serves as a versatile platform technology which could be easily adapted for specific applications, i.e. by exchanging the purification or antigen tags. We are currently focussing on how the modular secretion vector performs in terms of recovery of purified protein, free of the accessory tags.

## Conclusions

We have used a combinatorial approach, incorporating strain design and a genetic modular secretion vector, to achieve improved extracellular titres, of a range of biotechnologically relevant protein products, by secretion through a truncated *E. coli* FT3SS. This may enable simplified downstream processing of higher quality product, free of cellular contaminants, all of which are of great interest to the biopharmaceutical and IB communities. Furthermore, this opens up the possibility of cell cultures that secrete protein continuously, without the need to sacrifice cell culture to retrieve protein product. During development of the truncated secretion construct, a number of findings provided new information on the roles of the secretion signal peptide and UTRs of FliC monomers. We also introduce a high-throughput assay, enabling quick and accurate measurement of the secretion output of truncated FT3SS strains. We are currently using this in inverse genetic engineering approaches to identify factors that might boost secretion further. We suggest that the body of this work serves as a pilot feasibility study, and are working on further improvements (high cell density culture, purifying recombinant protein from the media), with which we envision that our FT3SS secretion platform could become a forerunner for use in the production of biopharmaceuticals and IB products.

## Materials and methods

### Bacterial strains and growth conditions

*E. coli* MC1000 was used for the construction of all mutants in this work (Additional file 1: Table S1). *E. coli* strain MG1655 *clpX::Tn5(Km*^*R*^*)* was kindly gifted by J. Green, University of Sheffield and was used as a donor for transduction in the construction of *clpX* strains. Strains were grown in LB broth or on LB agar plates, supplemented with ampicillin (100 µg mL^−1^), chloramphenicol (25 µg mL^−1^) or kanamycin (50 µg mL^−1^) or Isopropyl β-d-1-thiogalactopyranoside (IPTG) (concentration stated in figure legends) as appropriate. Prior to assaying protein secretion by SDS-PAGE or enzymatic secretion assay, fresh media was inoculated with cells from a liquid overnight culture and induced. Bacteria were grown with shaking at 180 rpm, 37 °C.

### P1 phage transduction

Phage transduction was carried out by the method outlined by Lennox [92]. Following strain construction, the eradication of phage from the strain was ensured by passaging on LB agar plates with 10 mM sodium citrate. Absence of phage was then confirmed by Evans Blue-Uranine assay, as outlined by Tiruvadi Krishnan et al. [93].

### Recombinant DNA techniques

Plasmids utilised for chromosomal manipulations or for cloning are listed in Additional file 1: Table S2. High fidelity and diagnostic polymerase chain reaction (PCR) was carried out with Phusion High Fidelity polymerase (New England Biosciences) and DreamTaq DNA Polymerase (Thermo Scientific) respectively. Templates and custom primers (Sigma-Aldrich) are listed in Additional file 1: Table S3. Chromosomal modifications were carried out, as described by Datsenko and Wanner [94]. To enable sequential mutagenesis, pCP20 was utilised to excise FRT flanked antibiotic resistance cassettes. Modifications were PCR verified, as was the maintenance of other lesions on the chromosome, if the strain underwent sequential deletions.

A genetic secretion construct was designed, synthesised and then incorporated into the pJexpress-404 plasmid backbone by DNA 2.0. This plasmid is referred to as pJex-*fliC*47-empty (Additional file 1: Fig. S4a) and forms the base cloning vehicle here. All restriction digests and ligation reactions were performed with NEB restriction enzymes and T4 DNA ligase (New England Biosciences). Following ligation, reaction mixes were transformed into NEB 5-alpha (New England Biosciences), as described in the supplier’s instructions. The synthetic gene for *Fusarium solani* cutinase (accession number: K02640), was codon optimised and synthesised by GeneArt^®^ Strings DNA Fragments (Life Technologies). hGH and TrxA gene fragments were obtained from FUJIFILM Diosynth Biotechnologies.

### SDS-PAGE and western immunoblots

Following cell growth for secretion, 1 OD unit of culture samples were centrifuged for 15 min (16,000*g*) to ensure separation of cells and supernatant. Supernatant fractions were prepared by precipitation in 10% v/v trichloroacetic acid and washing in acetone [57, 95]. Both supernatant precipitate and cell pellets were suspended in 2× SDS buffer (10 mL glycerol, 1 g SDS, 0.1 g bromophenol blue, 200 mM DTT, to a volume of 50 mL in 100 mM Tris–HCl, pH 6.8) to a final volume of 50 µL and 200 µL respectively. Both cell lysate and supernatant samples underwent SDS–polyacrylamide gel electrophoresis (SDS-PAGE). Gels were either stained with InstantBlue™ (Expedeon) or prepared for Western immunoblotting by electro-transfer onto nitrocellulose membrane (GE Healthcare Life Science) and blocked in 3% w/v bovine serum albumin in TBS (24 g L^−1^ Tris base, 88 g L^−1^ NaCl plus 0.1% v/v Tween). The following primary antibodies were diluted 1 in 1000 in TBS/Tween: H48 monospecific H rabbit antiserum (Statens Serum Institut), monoclonal ANTI-FLAG^®^M2 antibody produced in mouse (Sigma-Aldrich), *E. coli* GroEL rabbit IgG (Sigma-Aldrich). If required, following washing and re-blocking, anti-rabbit HRP (Cell Signalling Technology) was diluted 1 in 3000. All blocking and incubation steps were carried out for 1 h. Immunoblots were visualised using Pierce^®^ ECL Western Blotting Substrates (Thermo Scientific) and a Compact X4 X-ray Film Processor (Xograph) or C-DiGit^®^ Chemiluminescent Western Blot Scanner (LI-COR Biosciences). Analysis by densitometry was carried out using ImageJ. For FlhA detection, the same protocol was followed except blocking was carried out in 5% w/v skimmed milk in PBS (8 g L^−1^ NaCl, 0.2 g L^−1^ KCl, 1.44 g L^−1^ Na_2_HPO_4_, 0.24 g L^−1^ KH_2_PO_4_) with 0.05% v/v Triton X100. Polyclonal anti-FlhA antisera was diluted 1 in 1000 in PBS/Triton along with *E. coli* Δ*flhDC* soluble cell lysate (to minimise non-specific binding) and following washing, membranes were incubated with a 1 in 5000 dilution of IRDye 800CW Donkey anti-rabbit secondary antibody (LI-COR Biosciences). Membranes were analysed using an Azure c500 (Azure Biosystems) imaging system and quantified with LI-COR Image Studio Lite (LI-COR Biosciences).

### Protein purification for preparation of standards

To prepare purified monomers of truncated FliC, MC1000 Δ*fliC* harbouring pTrc-FliC-ΔDE3 were scraped from semisoft agar plates (1% w/v tryptone, 0.5% NaCl, 0.8% w/v bacteriological agar), following 24 h incubation at 37 °C. After resuspension in 50 mM Tris–HCl, pH 7.8, cells were agitated vigorously in a laboratory blender (Waring Laboratory Science) for 3 min, then centrifuged at 16,000 *g* for 5 min at 4 °C. The supernatant was then centrifuged at 67,500 *g* for 15 min at 4 °C. The pellet was resuspended in fresh buffer, and centrifugation repeated. In fresh buffer, the pellet underwent sonication for 20 s, followed by heating to 50 °C for 10 min to cause monomerisation. Finally, centrifugation was repeated and supernatant collected.

Protein standards of CH_2_, TrxA and hGH were purified from the intracellular fraction of *E. coli* BL21 (DE3) after induction with 1 mM IPTG. Protein was purified using the secretion construct incorporated Streptavadin II tag on a 1 mL StrepTrap™ HP column (GE Healthcare Life Science) according to the manufacturer’s instructions. Following elution with 2.5 mM desthiobiotin, 100 mM Tris–HCl, 150 mM NaCl, 1 mM EDTA, pH 8, protein underwent dialysis overnight to yield purified protein in 100 mM Tris–HCl, pH 8 and quantified with a Pierce™ BCA Protein Assay Kit (Thermo Scientific). Proteins standards were aliquoted and stored at − 20 °C.

### Cutinase based protein secretion assay

An assay based on cleavage of 4-methylumbelliferyl butyrate (MUB) was developed to measure the activity of secreted cutinase. 10 mL cultures of cells expressing cutinase were induced with 0.05 mM IPTG and grown to OD_600_ 1.0. 250 mM MUB (prepared fresh daily) was dissolved in dimethylformamide with 1% Triton X-100. This mixture was then diluted in 50 mM phosphate citrate (pH 5) buffer to a working concentration of 500 μM MUB. 40 μL supernatant was added to 96 well plate wells, the reaction was initiated with the addition of 160 μL MUB and incubated at 30 °C for 30 min. Following incubation, fluorescence was measured, either by imaging 96 well plates under UV light (G:BOX, Syngene), or using a Tecan Infinite 200 Pro plate reader-excitation 302 nm, emission 446 nm (Tecan Group Ltd.).

## Additional file

**Additional file 1: Table S1.** *Escherichia coli* strains used or generated in this study. **Table S2**. Plasmids used or generated in this study. **Table S3**. Polymerase chain reaction primers used in the study. **Fig. S1.** Annotation of the genetic region upstream of the *flhD* operon in *E. coli* MC1000. **Fig. S2.** Comparison of growth of the HAP-less and cap-less strains. Growth curve data for the aforementioned strains. **Fig. S3.** Functional flagellar despite the absence of the FliC D3 domain. SDS-PAGE of secreted protein and motility assay. **Fig. S4.** The effect of the deletion of *clpX* from *E. coli* MC1000. Comparison of the strains by: abundance of secreted flagellin, motility assay and phenotype. **Fig. S5.** Plasmid maps of (a) pJex-*fliC47*-empty and (b) pJex-*fliC47*-cutinase, along with (c) the prototype genetic synthetic modular secretion construct of pJex-*fliC47*-cutinase. **Fig. S6.** Comparison of growth of truncated FT3SS secretion strains. Growth curves for all relevant strains. **Fig. S7.** The FliC secretion signal is required to enable secretion. Immunoblots of secreted and intracellular cutinase. **Fig. S8.** Comparison of the ‘’late’ FliC and ‘early’ secretion signals. Schematic of secretion constructs and immunoblots of secreted and intracellular cutinase. **Fig. S9.** Secretion of a range of substrates through the optimised secretion strain. Immunoblots of secreted protein alongside a protein standard.

## Abbreviations

FT3SS: flagellar type III secretion system
T2SS: type II secretion system
IB: industrial biotechnology
MUB: 4-methylumbelliferyl butyrate
SC: secretion construct
TrxA: thioredoxin
hGH: human growth hormone
