# Supplementary material for: Engineering the flagellar type III secretion system: improving capacity for secretion of recombinant protein
Source: Microb Cell Fact. 2019 Jan 18;18:10. doi: 10.1186/s12934-019-1058-4 (PMC6337784; doi:10.1186/s12934-019-1058-4)
Supplement: Supplementary file 1 — Additional file 1: Table S1. Escherichia coli strains used or generated in this study. Table S2. Plasmids used or generated in this study. Table S3. Polymerase chain reaction primers used in the study. Fig. S1. Annotation of the genetic region upstream of the flhD operon in E. coli MC1000. Fig. S2. Comparison of growth of the HAP-less and cap-less strains. Growth curve data for the aforementioned strains. Fig. S3. Functional flagellar despite the absence of the FliC D3 domain. SDS-PAGE of secreted protein and motility assay. Fig. S4. The effect of the deletion of clpX from E. coli MC1000. Comparison of the strains by: abundance of secreted flagellin, motility assay and phenotype. Fig. S5. Plasmid maps of (a) pJex-fliC47-empty and (b) pJex-fliC47-cutinase, along with (c) the prototype genetic synthetic modular secretion construct of pJex-fliC47-cutinase. Fig. S6. Comparison of growth of truncated FT3SS secretion strains. Growth curves for all relevant strains. Fig. S7. The FliC secretion signal is required to enable secretion. Immunoblots of secreted and intracellular cutinase. Fig. S8. Comparison of the ‘’late’ FliC and ‘early’ secretion signals. Schematic of secretion constructs and immunoblots of secreted and intracellular cutinase. Fig. S9. Secretion of a range of substrates through the optimised secretion strain. Immunoblots of secreted protein alongside a protein standard. [file 12934_2019_1058_MOESM1_ESM.pdf]

Additional File 1

Additional tables

Additional File 1: Table S1. ***Escherichia coli*** **strains used or generated in this study.**

Strain	Relevant Genotype	Source
MC1000	F-, Δ*(araA-leu)7697, [araD139]$_{B/r}$*, Δ*(codB-lacI)3, galK16, galE15*(GalS), *λ-, e14-, relA1, rpsL150* (strR), *spoT1, mcrB1*	[1]
MG1655 *clpX::Tn5(Km^R)*	F-, *λ* -, rph-1, *clpX::Km(Tn5)*	J. Green, University of Sheffield
NEB DH5α	*fhuA2 lac(del)U169 phoA glnV44 Φ80' lacZ(del)M15 gyrA96 recA1 relA1 endA1 thi$_{-1}$ hsdR17*	New England Biotechnologies
BL21 (DE3)	*fhuA2 [lon] ompT gal (λ DE3) [dcm] ΔhsdS λ DE3 = λ sBamHIo ΔEcoRI-B int::(lacI::PlacUV5::T7 gene1) i21 Δnin5*	New England Biotechnologies
Δ*flhD(C)*	MC1000 Δ*flhD::*FRT-*Km^R* –FRT	[2]
Δ*fliC*	MC1000 Δ*fliC::*FRT-*Km^R* –FRT	This study
Δ*fliCD*	MC1000 Δ*fliCD::*FRT-*Km^R* –FRT	This study
Δ*fliC* Δ*flgKL* Δ*fliCD*	MC1000 Δ*fliC* Δ*flgKL* Δ*fliCD::*FRT-*Km^R* –FRT	This study
Δ*fliC* Δ*flgKL:: Km^R*	*MC1000* Δ*fliC* Δ*flgKL::*FRT-*Km^R* –FRT	This study
Δ*fliC* Δ*flgKL*	*MC1000* Δ*fliC* Δ*flgKL::FRT*	This study
Δ*fliC* Δ*flgKL* Δ*clpX*	MC1000 Δ*fliC* Δ*flgKL::*FRT *clpX::Tn5(Km^R)*	This study
Δ*fliC* Δ*flgKL* Δ*fliDST*	MC1000 Δ*fliC* Δ*flgKL::*FRT Δ*fliDST::*FRT-*Km^R* -FRT	This study
Δ*fliC* Δ*flgKL* Δ*flgMN*	MC1000 Δ*fliC* Δ*flgKL::*FRT Δ*flgMN::*FRT-*Km^R* -FRT	This study
Δ*fliC* Δ*flgKL* Δ*motAB*	MC1000 Δ*fliC* Δ*flgKL::*FRT Δ*motAB::*FRT-*Km^R* -FRT	This study
Δ*fliC* Δ*flgKL* Δ*fliDST* Δ*flgMN*	MC1000 Δ*fliC* Δ*flgKL::*FRT Δ*fliDST::*FRT-*Cm^R* -FRT Δ*flgMN::*FRT-*Km^R* –FRT	This study

Δ*fliC* Δ*flgKL* Δ*motAB* Δ*fliDST*	MC1000 Δ*fliC* Δ*flgKL::*FRT Δ*motAB::*FRT-*Cm*^{R}-FRT Δ*fliDST::*FRT-*Km*^{R}–FRT	This study
Δ*fliC* Δ*flgKL* Δ*motAB* Δ*flgMN*	MC1000 Δ*fliC* Δ*flgKL::*FRT Δ*motAB::*FRT-*Cm*^{R}-FRT Δ*flgMN::*FRT-*Km*^{R}–FRT	This study
Δ*fliC* Δ*flgKL* Δ*clpX* Δ*fliDST*	MC1000 Δ*fliC* Δ*flgKL::*FRT *clpX::Tn5(Km*^{R}*)* Δ*fliDST::*FRT-*Cm*^{R}-FRT	This study
Δ*fliC* Δ*flgKL* Δ*clpX* Δ*flgMN*	MC1000 Δ*fliC* Δ*flgKL::*FRT *clpX::Tn5(Km*^{R}*)* Δ*flgMN::*FRT-*Cm*^{R}-FRT	This study
Δ*fliC* Δ*flgKL* Δ*clpX* Δ*motAB*	MC1000 Δ*fliC* Δ*flgKL::*FRT *clpX::Tn5(Km*^{R}*)* Δ*motAB::*FRT-*Cm*^{R}-FRT	This study
Δ*fliC* Δ*flgKL* Δ*clpX* Δ*motAB* Δ*fliDST*	MC1000 Δ*fliC* Δ*flgKL::*FRT *clpX::Tn5(Km*^{R}*)* Δ*motAB::*FRT Δ*fliDST::*FRT-*Cm*^{R}-FRT	This study
Δ*fliC* Δ*flgKL* Δ*clpX* Δ*motAB* Δ*fliDST*	MC1000 Δ*fliC* Δ*flgKL::*FRT *clpX::Tn5(Km*^{R}*)* Δ*motAB::*FRT Δ*flgMN::*FRT-*Cm*^{R}-FRT	This study
Δ*fliC* Δ*flgKL* Δ*flgDE*	MC1000 Δ*fliC* Δ*flgKL::*FRT Δ*flgDE::*FRT-*Km*^{R}-FRT	This study
Δ*fliC* Δ*flgKL* Δ*flgDE* Δ*clpX*	MC1000 Δ*fliC* Δ*flgKL::*FRT Δ*flgDE::*FRT *clpX::Tn5(Km*^{R}*)*	This study

Additional File 1: Table S2. Plasmids used or generated in this study.

Plasmid	Description/function	Source
pTrc99a-FF	Empty vector for protein expression. pTrc99a with *Nde*I site in multiple cloning region. Ap^R	Gillian Fraser, UoC. [3]
pTrc99a-FliC-FF	*E. coli fliC* inserted between *Nde*I and *Bam*HI of pTrc99a-FF. Ap^R	Gillian Fraser, UoC
pTrc99a-FliC-ΔDE3	*E. coli fliC* Δ191-283 inserted between *Nde*I and *Bam*HI of pTrc99a-FF. Ap^R	Dr. Matthew Hicks, UoS
pJex-*fliC*47-empty	pJexpress-404-1-47 *fliC* secretion construct. pJexpress 404 with *Nde*I- *fliC* 5'UTR- secretion signal residues 1-47- *Xho*I- TEV- *Eco*R1- *Pst*I- TEV- FLAG tag- Step tag-*Xba*I- *Hind*III- *fliC* 3'UTR- *Bam*HI. Ap^R	DNA 2.0, USA
pJex-*fliC*47-CH2	Derivative of pJex-*fliC*47-empty with CH2 inserted between *Eco*RI and *Pst*I. Ap^R	Dr. Matthew Hicks, UoS, CH2: Dr Jagroop *Pandhal*
pSC0: pJex-*fliC*47–cutinase	Synthetic *F. solani* cutinase (accession: K02640.1) gene inserted between *Eco*RI and *Pst*I of pJex-*fliC*47-empty. Ap^R	This study
pSC1: pJex-*fliC*47–cutinase-no 3'UTR	Template was PCR amplified pSC0 using primers with *Nde*I and *Bam*HI extensions and then inserted between *Nde*I and *Bam*HI of pJex-*fliC*47-empty. Ap^R	This study
pSC2: pJex-*fliC*47–cutinase-no 5'UTR	Template was PCR amplified from pSC0 using primers with *Nde*I and *Bam*HI extensions and then inserted between *Nde*I and *Bam*HI of pJex-*fliC*47-empty. Ap^R	This study

pSC3: pJex-*fliC*47–cutinase-no 5'UTR or 3'UTR	Template was PCR amplified from pSC0 using primers with *Nde*I and *Bam*HI extensions and then inserted between *Nde*I and *Bam*HI of pJex-*fliC*47-empty. Ap^R	This study
pSC4: pJex-*fliC*26-47-cutinase-no 5'UTR	Template was PCR amplified from pSC0 using primers with *Nde*I and *Bam*HI extensions and then inserted between *Nde*I and *Bam*HI of pJex-*fliC*47-empty. Ap^R	This study
pSC5: pJex-*fliC*26-47-cutinase-no 5'UTR or 3' UTR	Template was PCR amplified pSC0 using primers with *Nde*I and *Bam*HI extensions and then inserted between *Nde*I and *Bam*HI of pJex-*fliC*47-empty. Ap^R	This study
pSC6: pJex-*Sal fliC*26-47-cutinase-no 5'UTR	Template was PCR amplified from pSC0 using primers with *Nde*I and *S. typhimurium* 26-28 residue and *Bam*HI extensions and then inserted between *Nde*I and *Bam*HI of pJex-*fliC*47-empty. Ap^R	This study
pSC7: pJex-*Sal fliC*26-47-cutinase-no 5'UTR or 3'UTR	Template was PCR amplified from pSC0 using primers with *Nde*I and *S. typhimurium* 26-28 residue and *Bam*HI extensions and then inserted between *Nde*I and *Bam*HI of pJex-*fliC*47-empty. Ap^R	This study
pSC8: pJex-*fliC*-cutinase-no 5'UTR or *fliC*47	Template was PCR amplified from pSC0 using primers with *Nde*I and *Bam*HI extensions and then inserted between *Nde*I and *Bam*HI of pJex-*fliC*47-empty. Ap^R	This study
pSC9: pJex-cutinase-no *fliC*47, 5'UTR or 3'UTR	Template was PCR amplified from pSC0 using primers with *Nde*I and *Bam*HI extensions and then inserted between *Nde*I and *Bam*HI of pJex-*fliC*47-empty. Ap^R	This study
pSC10: pJex-*fliC*-cutinase-no *fliC*47	The 5'UTR of *fliC* was PCR amplified from *E. coli* MC1000 using primers with *Nde*I and *Xho*I extensions and then inserted between *Nde*I and *Xho*I of pSC0. Ap^R	This study
pSC11: pJex-*fliC*-cutinase-no *fliC*47 or 3'UTR	The 5'UTR of *fliC* was PCR amplified from *E. coli* MC1000 using primers with *Nde*I and *Xho*I extensions and then inserted between *Nde*I and *Xho*I of pSC1. Ap^R	This study
pSC12: pJex-*flgE*100–cutinase-no 5'UTR or 3'UTR	The 100 amino acid N-terminal of *flgE* was PCR amplified from *E. coli* MC1000 using primers with *Nde*I and *Xho*I extensions and then inserted between *Nde*I and *Xho*I of pSC1. Ap^R	This study
pSC13: pJex-*flgD*100-cutinase-no 3'UTR	The 100 amino acid N-terminal of *flgD* was PCR amplified from *E. coli* MC1000 using primers with *Nde*I and *Xho*I extensions and then inserted between *Nde*I and *Xho*I of pSC0. Ap^R	This study_
pJex-*fliC*47–CH2 (no 3'UTR)	CH2 from pJex-*fliC*47 -CH2 PCR amplified with *Eco*RI and *Pst*I sites and inserted between *Eco*RI and *Pst*I of pSC1. Ap^R	This study
pJex-*fliC*47–hGH (no 3'UTR)	hGH from FUJIFILM Diosynth Biotechnologies, UK gene amplified with *Mfe*I and *Nsi*I sites and inserted between *Eco*RI and *Pst*I of pSC1. Ap^R	This study
pJex-*fliC*47–TrxA (no 3'UTR)	TrxA from FUJIFILM Diosynth Biotechnologies, UK gene amplified with *Mfe*I and *Nsi*I sites and inserted between *Eco*RI and *Pst*I of pSC1. Ap^R	This study
pKD46	Expression of λ-RED recombinase system for recombineering. Ap^R	Barry Wanner, USA
pCP20	Expression of FLP recombinase for excision of FRT-flanked markers. Ap^R	Barry Wanner, USA
pKD3	Template for overlap PCR and cassette construction.	Barry Wanner, USA

	Cm^R	
pKD4	Template for overlap PCR and cassette construction. Km^R	Barry Wanner, USA
pKD13	Template for overlap PCR and cassette construction. Km^R	Barry Wanner, USA
pKD32	Template for overlap PCR and cassette construction. Cm^R	Barry Wanner, USA

Additional File 1: Table S3. Polymerase chain reaction primers used in the study.

Primer	Nucleotide Sequence	Function
**K1**	CAGTCATAGCCGAATAGCCT	Conformation of gene knockout
**K2**	CGGTGCCCTGAATGAACTGC	Conformation of gene knockout
**C1**	TTATACGCAAGGCGACAAGG	Conformation of gene knockout
**C2**	GATCTTCCGTCACAGGTAGG	Conformation of gene knockout
**K1**	CAGTCATAGCCGAATAGCCT	Conformation of gene knockout
**motAB KO FOR**	GCCTGACGACTGAACATCCTGTCATGGTCAACAGT GGAAGGATGATGTCGTGTAGGCTGGAGCTGCTTC	Construction of gene knockout cassettes
**motAB KO REV**	TTCATCAAAAAATGTCTGATAAAAATCGCTTATATC CATGCTCACGCTGCATATGAATATCCTCCTTA	Construction of gene knockout cassettes
**motAB KO CONFO**	ATTTTATTACCCACGCTCACCAGCCTGTTGGC	Conformation of gene knockout
**flgMN KO FOR**	CGATAAATAAGCAACACATGATAAAAGCGCCCTCA ATGAGGAATAAACCGTGTAGGCTGGAGCTGCTTC	Construction of gene knockout cassettes
**flgMN KO REV**	GACGGTGTAACAATGCATTCCGGCCTGCAGTGCAG GCCGGAGATAATCTCATATGAATATCCTCCTTA	Construction of gene knockout cassettes
**flgMN KO CONFO**	TAACCCAGTTTCGCCAGGCATGGCGGGTAAAA	Conformation of gene knockout
**flgDE KO F**	AGCATGATGCTGAAAACCCTTACGCTCGGTCAATA AAGGAGAAAGCTGTGTAGGCTGGAGCTGCTTC	Construction of gene knockout cassettes
**flgDE KO R**	TCGCGGTATAAATTGCGTGATCCATTGAGCTATCCC GTCAGCGACATATGAATATCCTCCTTA	Construction of gene knockout cassettes
**flgDE KO CONFO**	ATTCCAGGTTAACGCTGCACCAGGTGCTGCGACAG GCGG	Conformation of gene knockout
**fliDST KO FOR**	CGATAACCCCGGTATTCGTTTTACGTGTCGAAAGAT AAAAGGAAATCGCGTGTAGGCTGGAGCTGCTTC	Construction of gene knockout cassettes
**fliDST KO REV**	GAAGCGTAGCCGTAATCGGATTATTCGCGAGCCAT CGACTCATTCAGATATTCCGGGGATCCGTCGAC	Construction of gene knockout cassettes
**fliDST KO CONFO**	TGACTTGTGCCATGATTCGTTATCCTATATTG	Conformation of gene knockout
**5’ and 47aa present**	CGGTACCATATGGCGGGAATAAGGGGCAGAG	Construction of secretion

**(5' UTR 47aa F)**		construct variants
**No 5', 47aa present (47aa F)**	CGGTGACATATGAAAGAGGAGAAATAGTCCATGG CACAAGTCATTAATACCAACAGCCTCTC	Construction of secretion construct variants
**No 5' or 47aa**	TTAACTTCATATGCTCGAGAGGATAACGAATCATG GAGAATCTGTATTTTCAGGGCGAATCC	Construction of secretion construct variants
**coli 26-47 F**	CGGTACCATATGAAAGAGGAGAAATAGTCCATGTC GAGTTCTATCGAGCGTCTGTCTTCTGGCTTGCG	Construction of secretion construct variants
**sal 26-47 F**	CGGTACCATATGAAAGAGGAGAAATAGTCCATGG GAACGGCAATCGAGCGTCTGTCTTCTGGC	Construction of secretion construct variants
**3' UTR present (3' UTR R)**	TACAGAGGATCCCACGATAAACAGCCCTGCGTT	Construction of secretion construct variants
**3' UTR absent (no 3')**	CGCAGTTTGAGAAATCTAGAAAGCTTTAAGGATCC CGTTAG	Construction of secretion construct variants
**coli 26-47 F**	CGGTACCATATGAAAGAGGAGAAATAGTCCATGTC GAGTTCTATCGAGCGTCTGTCTTCTGGCTTGCG	Construction of secretion construct variants
**sal 26-47 F**	CGGTACCATATGAAAGAGGAGAAATAGTCCATGG GAACGGCAATCGAGCGTCTGTCTTCTGGC	Construction of secretion construct variants
**pJex ins F**	CGAGCGTCTGTCTTCTGGCTTGCGTATTAACAG	Colony PCR and sequencing of inserts in pJexpress
**pJex seq F**	AACGGTTTCCCTCTAGAAATAATTTTGTTT	Colony PCR and sequencing of inserts in pJexpress
**pJex seq R**	TTCGCCCGGGCTAATTATGGGGTGTCGCCC	Colony PCR and sequencing of inserts in pJexpress
**pJex seq alt F**	ATTCCACAACGGTTTCCCTCTAGAAATAATTTTG	Colony PCR and sequencing of inserts in pJexpress

Additional Figures

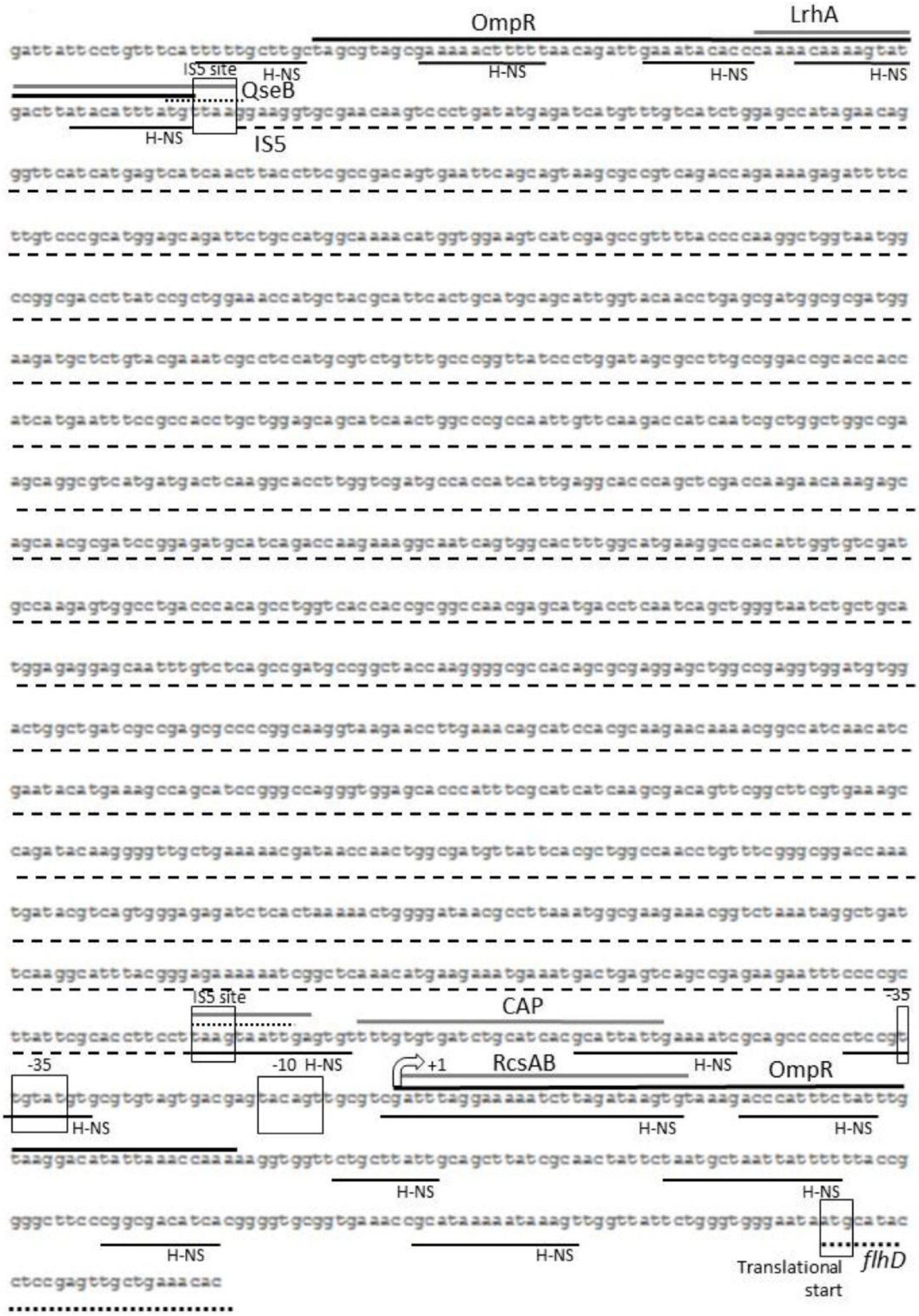

Additional File 1: Fig. S1. Annotation of the genetic region upstream of the *flhD* operon in *E. coli* MC1000. Based on sequencing of the whole genome by MicrobesNG, University of Birmingham. Binding sites for regulatory proteins shown for: H-NS (histone-like nucleoid-structuring protein), OmpR, LrhA, CRP (cAMP-receptor protein), RscAB and QseB. The *flhD* gene, transcriptional start site (+1) of the *flhD* operon and the -10 and -35 promoter sequences are also labelled. Also shown is the IS5 insertion (dashed line) and duplication of the IS5 insertion site to flank the IS5 element [4–6].

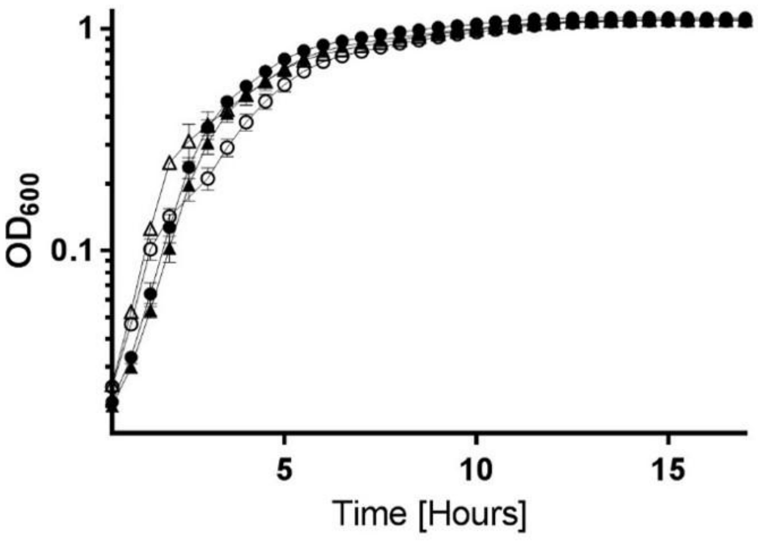

Additional File 1: Fig. S2. Comparison of growth of the HAP-less and cap-less strains. *E.coli* MC1000 Δ*fliC* Δ*flgKL* and Δ*fliC*D cells containing pTrc-FliC-ΔD3 or pTrc-empty were supplemented with 0.05 mM IPTG and grown in 96 well plate wells, with incubation in a

TECAN plate reader at 37°C with 6mm orbital shaking, OD_{600} measurements were recorded every 30 minutes. Three technical repeats of two biological repeats, ± SE. Δ*fliC* Δ*flgKL* + pTrc-FliC-ΔD3: ▲, Δ*fliC* Δ*flgKL* + pTrc-empty: ⍰, Δ*fliCD* + pTrc-FliC-ΔD3: ●, Δ*fliCD* + pTrc-empty: ○.

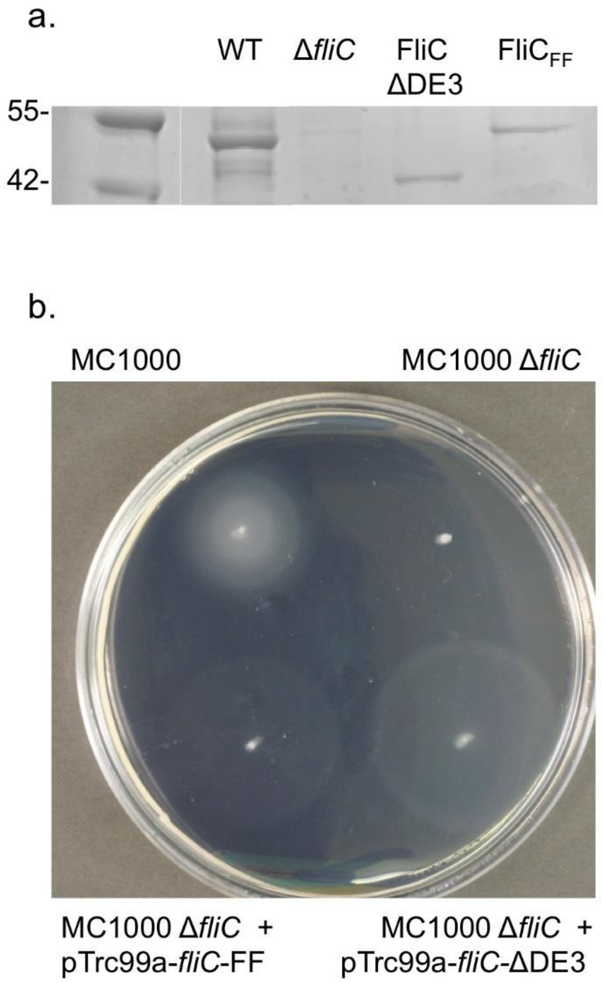

Additional File 1: Fig. S3. Functional flagellar despite the absence of the FliC D3 domain. Secreted FliC and motility in MC1000 wildtype, Δ*fliC,* Δ*fliC* + pTrc-FliC-ΔD3 and Δ*fliC* + pTrc-FliC-FF (a) Coomassie stained SDS-PAGE of the secreted fraction, showing the presence of FliC protein in wildtype MC1000, along with the shortened FliC-ΔD3 and full size FliC

monomer in a Δ*fliC* background. (b) Swimming motility of cells on 0.25% LB agar plates, during incubation at 30°C.

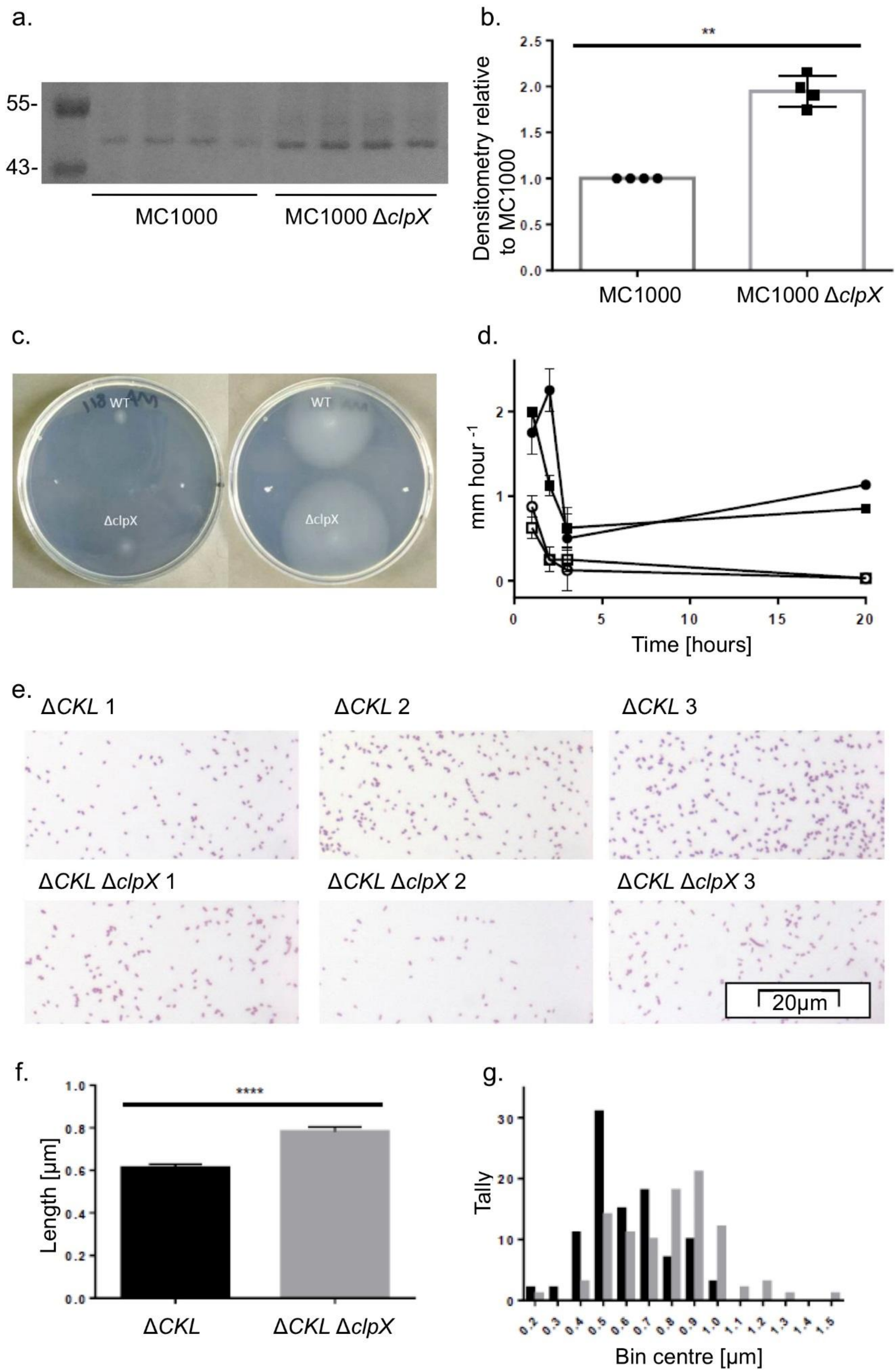

Additional File 1: Fig. S4. The effect of the deletion of *clpX* from *E. coli* MC1000. (a)

Following cell culture of ΔCKL or ΔCKL Δ*clpX* in LB media, cells were harvested and normalised for OD_{600}. Filaments were isolated and loaded on to an SDS-PAGE for Coomassie staining. (b) The relative abundance of filament protein was calculated by densitometry analysis and normalisation to MC1000. SE ± for four biological replicates displayed, two-way t-test: ** = p <0.01. (c) Swimming motility of (clockwise from the top) MC1000 (WT), MC1000 Δ*fliC* Δ*flgKL,* MC1000 Δ*clpX* and MC1000 Δ*fliC* Δ*flgKL* Δ*clpX* cells on 0.25% LB agar plates, during incubation at 30°C. Images were recorded following 1 hour (left) and 24 hours (right). (d) Swimming motility was measured at time intervals during incubation, allowing swimming speed to be calculated. MC1000: ■, MC1000 Δ*fliC* Δ*flgKL:* □, MC1000 Δ*clpX*: ● and MC1000 Δ*fliC* Δ*flgKL* Δ*clpX:* ○. Four biological replicates, ± SE. (e) Gram stain images (100x magnification) of heat fixed cells following growth in LB. (f) Average length of cells ± SE. **** = p <0.001. Unpaired t-test. N = 99 (Three biological replicates, 33 measurements from each). (g) Results from (f) sorted into bins for length (0.1µM centres). Black: ΔCKL. Grey: ΔCKL Δ*clpX*

a.

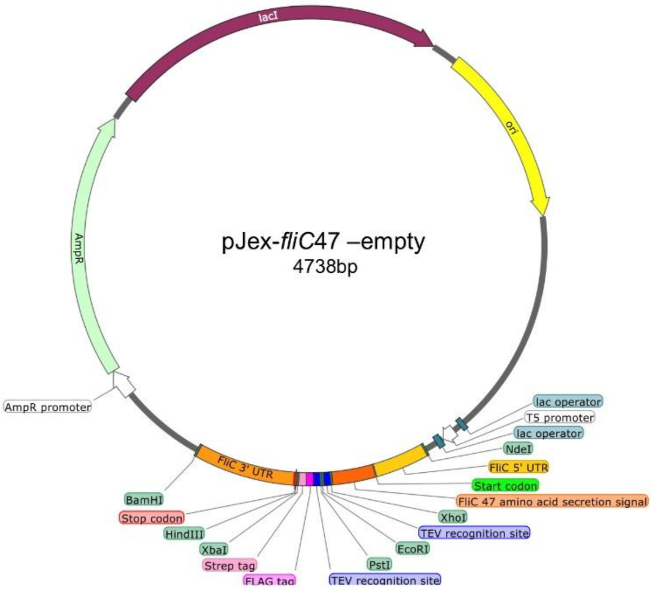

b.

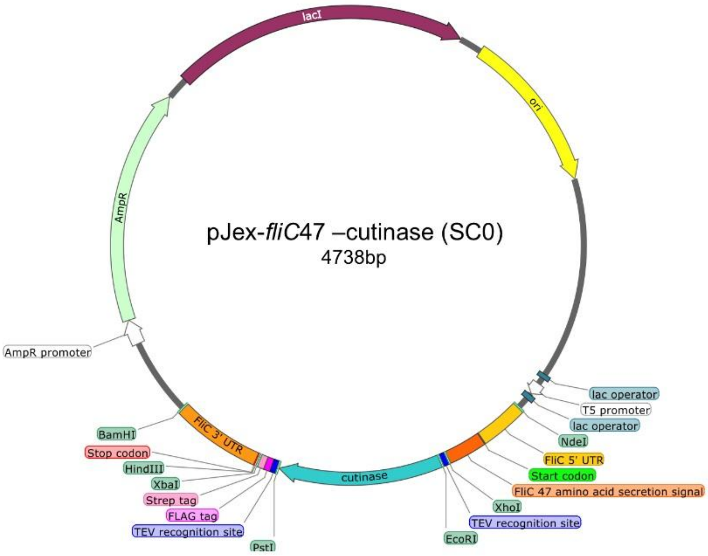

c. Genetic prototype secretion construct (SC0)

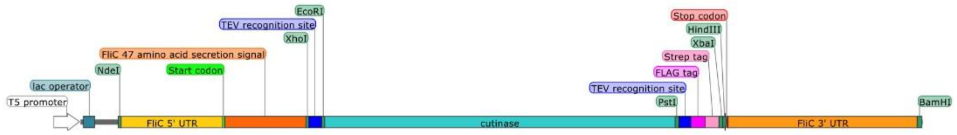

Additional File 1: Fig. S5. Plasmid maps of (a) pJex-*fliC47*–empty and (b) pJex-*fliC47*–cutinase, along with (c) the prototype genetic synthetic modular secretion construct of pJex-*fliC47*–cutinase. The genomic prototype modular secretion construct is a *trc* based

IPTG inducible construct harbouring the gene for *F. solani* cutinase along with the 5' and 3' FliC UTRs, the 47 peptide FliC secretion signal, a FLAG and a streptavidin II tag for purification and detection with antibodies and TEV protease cleavage sites to yield pure protein product. Restriction enzyme sites are incorporated throughout to allow modification of the secretion construct in a modular fashion. The prototype secretion signal construct is under transcriptional control of the IPTG inducible T5 promoter and the sigma factor-28 (FliA) inducible *fliC* promoter which is harboured in the 5' UTR. Secretion construct variants were constructed by PCR amplification of the required nucleotides from pJex-*fliC*–cutinase. Primers were designed with extensions to allow inclusion of *Nde*I or *Bam*HI. If necessary, a ribosome binding site, start or stop codon and any additional modifications (*i.e.* the presence of *S. typhimurium* secretion signal residues 26-28) were also included. PCR derived templates and pJex-*fliC*47–empty underwent restriction digest with *Nde*I and *Bam*HI, and were ligated and sequence verified prior to expression.

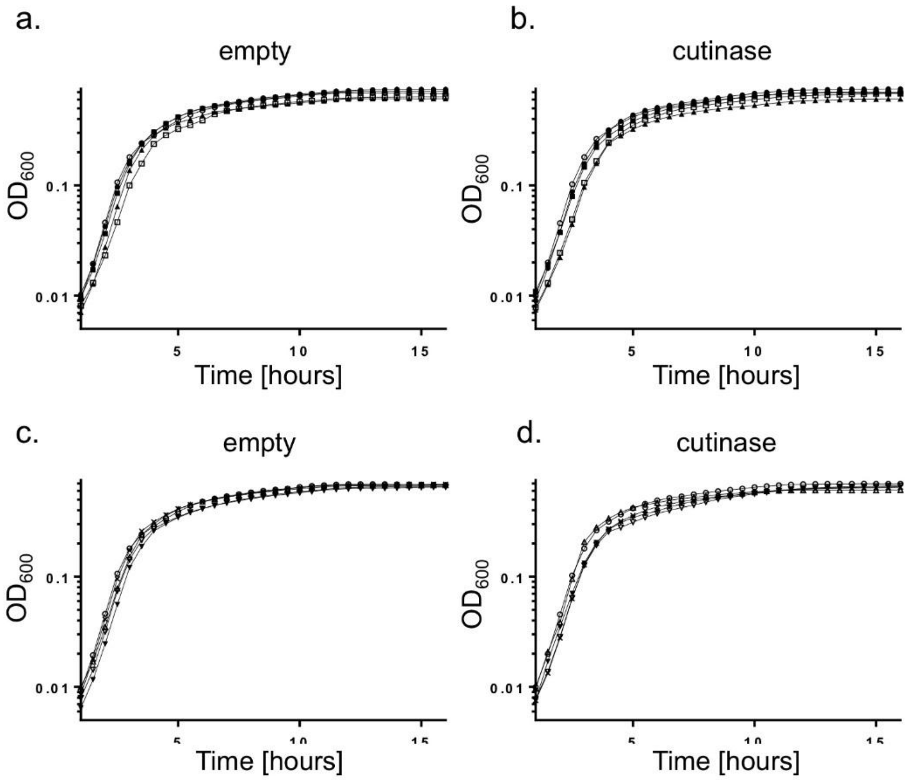

Additional File 1: Fig. S6. Comparison of growth of truncated FT3SS secretion strains. *E. coli* MC1000 Δ*fliC* Δ*flgKL* (ΔCKL) and strains with additional mutations containing pJex-*fliC*47–cutinase or pJex-*fliC*47–empty were grown as described in Fig. S2. Results split into four graphs for ease of viewing with Δ*fliC* Δ*flgKL* and the relevant plasmid on all graph to allow comparison. Three technical repeats of two biological repeats, ± SE. (a, c) ΔCKL: ○, ΔCKL Δ*flgMN* Δ*fliDST*: ●, ΔCKL Δ*motAB* Δ*flgMN:* ■*,* ΔCKL Δ*motAB* Δ*fliDST:* □*,* ΔCKL Δ*clpX* Δ*motAB:* ▲. (b, c) B and D: ΔCKL: ○, ΔCKL Δ*clpX* Δ*flgMN:* ⍰, ΔCKL Δ*clpX* Δ*fliDST:* ▼*,* ΔCKL Δ*clpX* Δ*motAB* Δ*flgMN:* and ΔCKL Δ*clpX* Δ*motAB* Δ*fliDST:* X.

a.

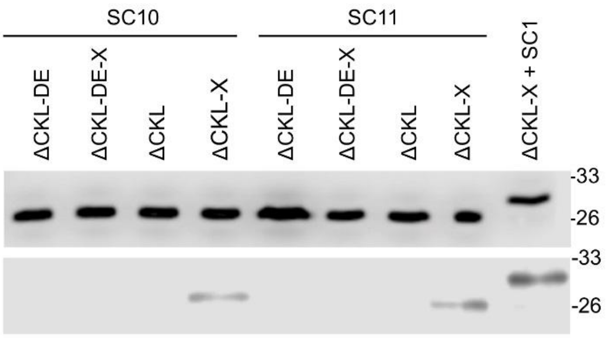

b.

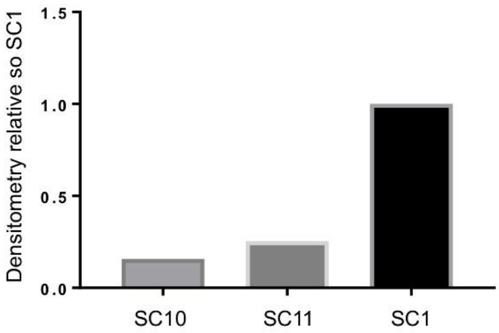

Additional File 1: Fig. S7. The FliC secretion signal is required to enable secretion. (a) Secretion profiles of SC10 and SC11 (as shown in Fig. 6) which harbour the 5'UTR, but lack the 47aa FliC secretion signal (and in the case of SC11, the 3'UTR), in early locked (Δ*fliC* Δ*flgKL* Δ*flgDE* (Δ*clpX*)) or substrate switched (Δ*fliC* Δ*flgKL* (Δ*clpX*) backgrounds, along with SC1 expressed in Δ*fliC* Δ*flgKL* Δ*clpX,* to allow comparison. Cells were grown as described in Fig. 3. Both intracellular and secreted fractions underwent immunoblot analysis using an anti-FLAG-HRP antibody to detect intracellular and secreted cutinase. Samples representing 25 µL and 1350 µL cell culture were loaded for intracellular and supernatant samples, respectively. (b) Densitometry and normalisation (to SC1) of the secreted fractions of Δ*fliC* Δ*flgKL* Δ*clpX* cells shown in (a).

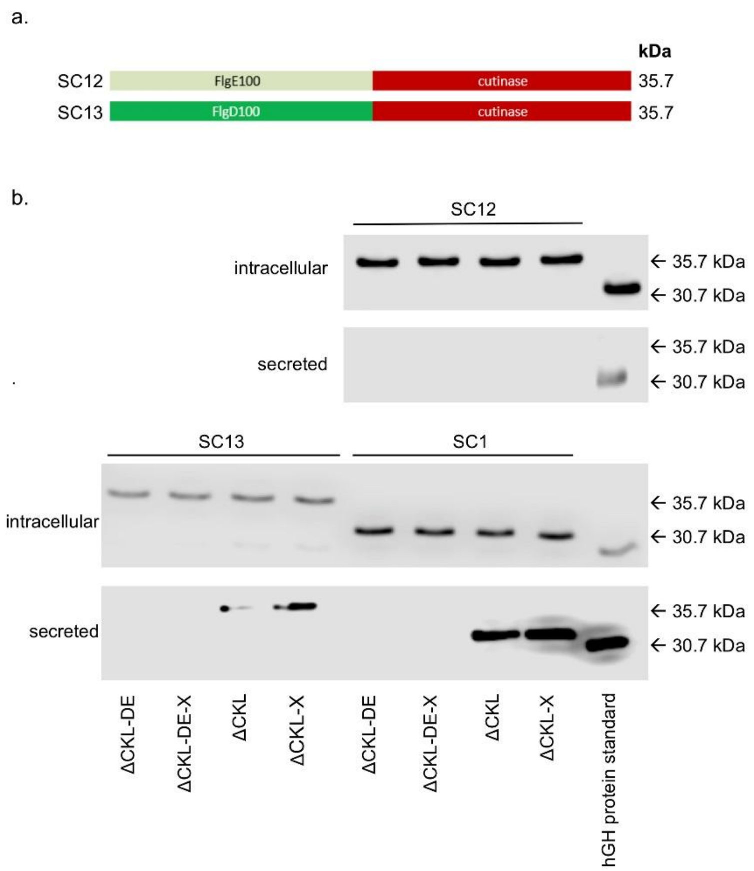

Additional File 1: Fig. S8. Comparison of the 'late' FliC and 'early' secretion signals. (a) Schematic of the 'early' secretion signal construct variants and the predicted size of the protein product (kDa). All secretion constructs harbour cutinase, along with 100 residue secretion tag of FlgE (SC12) or FlgD (SC13). Construction of the corresponding plasmids is outlined in Fig. S5 and Table S2. (b) Secretion profiles of SC12 and SC13, along with SC1 for comparison, in early locked (Δ*fliC* Δ*flgKL* Δ*flgDE* (Δ*clpX*)) or substrate switched (Δ*fliC* Δ*flgKL* (Δ*clpX*) backgrounds. Cells were grown as described in Fig. 3. Both intracellular and secreted fractions underwent immunoblot analysis using an anti-FLAG-HRP antibody to detect intracellular and secreted cutinase. A hGH protein standard was also loaded to allow quantification and serve as a positive control. Samples representing 25 µL and 1350 µL cell culture were loaded for intracellular and supernatant samples, respectively.

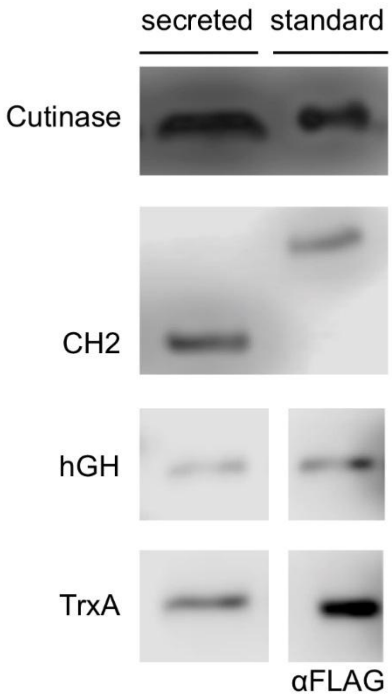

Additional File 1: Fig. S9. Secretion of a range of substrates through the optimised secretion strain. *E.coli* Δ*fliC* Δ*flgKL* Δ*clpX* Δ*motAB* expressing plasmid based SC1 with either cutinase, CH2, hGH or TrxA cargo were grown as outlined in Fig. 3 and prepared, along with a relevant protein standard (with the exception of cutinase, where a hGH standard was utilised), for immunoblot detection with anti-FLAG-HRP (αFLAG).

Additional File 1: References

1. Casadaban MJ, Cohen SN. Analysis of gene control signals by DNA fusion and cloning in Escherichia coli. J Mol Biol. 1980;138:179–207. doi:10.1016/0022-2836(80)90283-1.

2. Prüss BM, Campbell JW, Van Dyk TK, Zhu C, Kogan Y, Matsumura P. FlhD/FlhC is a regulator of anaerobic respiration and the Entner-Doudoroff pathway through induction of the methyl-accepting chemotaxis protein Aer. J Bacteriol. 2003;185:534–43. doi:10.1128/JB.185.2.534-543.2003.

3. Oshima T, Ishikawa S, Kurokawa K, Aiba H, Ogasawara N. Escherichia coli histone-like protein H-NS preferentially binds to horizontally acquired DNA in association with RNA polymerase. DNA Res. 2006;13:141–53. doi:10.1093/dnares/dsl009.

4. Yanagihara S, Iyoda S, Ohnishi K, Iino T, Kutsukake K. Structure and transcriptional control of the flagellar master operon of Salmonella typhimurium. Genes Genet Syst. 1999;74:105–11. http://www.ncbi.nlm.nih.gov/pubmed/10586519. Accessed 30 Aug 2016.

5. Barker CS, Prüss BM, Matsumura P. Increased motility of Escherichia coli by insertion sequence element integration into the regulatory region of the flhD operon. J Bacteriol. 2004;186:7529–37. doi:10.1128/JB.186.22.7529-7537.2004.

6. Clarke MB, Sperandio V. Transcriptional regulation of flhDC by QseBC and sigma (FliA) in enterohaemorrhagic Escherichia coli. Mol Microbiol. 2005;57:1734–49. doi:10.1111/j.1365-2958.2005.04792.x.
